# CD109 Drives Stemness and Chemoresistance in Colorectal Cancer via LRRC8A/AKAP12/PKCα-Mediated STAT3 Activation and WNT Signaling

**DOI:** 10.7150/ijbs.127856

**Published:** 2026-06-04

**Authors:** Rubén A. Bartolomé, Tania Calvo-López, Pablo Otero-Núñez, María Estaras, Asunción Fernández-Barral, Antonio Barbáchano, Issam Boukich, María Cuerda-López, Mª Jesús Fernández-Aceñero, Gorane Rodríguez-Urquirizar, Ana Mariscal-Casero, Miguel Padilla-Blanco, María Montoya, Ana O'Loghlen, José M. Fernández-Fernández, Jose Manuel Gonzalez-Sancho, J. Ignacio Casal

**Affiliations:** 1 Department of Biomedicine. Centro de Investigaciones Biológicas (CIB-CSIC). Ramiro de Maeztu 9, 28040 Madrid, Spain.; 2 Instituto de Investigaciones Biomédicas Sols-Morreale, Consejo Superior de Investigaciones Científicas - Universidad Autónoma de Madrid, 28049 Madrid, Spain.; 3 Departamento de Bioquímica, Facultad de Medicina, Universidad Autónoma de Madrid, 28029 Madrid, Spain.; 4 Centro de Investigación Biomédica en Red de Cáncer (CIBERONC), Spain.; 5 Instituto de Investigación Sanitaria del Hospital Universitario La Paz-IdiPAZ, 28029 Madrid, Spain.; 6 Protein Alternatives SL. Tres Cantos, Madrid, Spain.; 7 Pathology Department. Hospital Clínico San Carlos (HCSC), Madrid, Spain.; 8 Laboratori de Fisiologia Molecular, Departament de Medicina i Ciències de la Vida, Universitat Pompeu Fabra, 08003 Barcelona, Spain.

**Keywords:** CD109, LRRC8A, AKAP12, STAT3, TGFβ signaling, Wnt signaling, chemoresistance, metastasis, colorectal cancer

## Abstract

Colorectal cancer (CRC) is the third most prevalent type of cancer worldwide, with a poor survival rate at the metastatic stage. Here, we identify CD109—a negative regulator of TGFβ signaling—as a key driver of stemness and drug resistance through modulation of Wnt signaling in advanced CRC. CD109 expression strongly correlates with TGFβ levels in patient tumors and is enriched in the aggressive CRIS-B subtype, where it associates with poor clinical outcome. CD109 silencing reduced STAT3 phosphorylation and cell proliferation, without affecting migration or invasion. Moreover, global expression analysis revealed downregulation of various hallmarks of cancer stemness (i.e. LGR5 expression), together with increased TGFβ signaling and cellular senescence. Mechanistically, CD109 interacts with LRRC8A, a subunit of the volume-regulated anion channel (VRAC), which associates with AKAP12 to activate PKCα and promote STAT3 phosphorylation. This CD109/LRRC8A/AKAP12/PKCα axis sustains Wnt signaling, stemness, and drug resistance. Consistently, co-expression of CD109/LRRC8A/AKAP12 correlates with poor prognosis in CRC patients. Genetic or pharmacological disruption of this CD109/LRRC8A/AKAP12/PKCα axis impaired STAT3 signaling, reduced LGR5 expression and Wnt signaling, and sensitized cells to chemotherapy. *In vivo*, CD109 or LRRC8A knockdown significantly impaired liver homing and metastatic colonization in mouse models, showing stronger effects in Swiss nude mice than in highly immunodeficient NSG mice. Collectively, these findings support CD109 as a central regulator for STAT3-driven stemness and chemoresistance in advanced CRC, via the LRRC8A/AKAP12/PKCα axis, and highlight its potential value as a therapeutic target in metastatic disease.

## Introduction

Colorectal cancer (CRC) metastasis is a major cause of cancer-associated death in developed countries. Metastatic cells have to survive in a harsh and different microenvironment, and evade the anti-proliferative signals from Transforming Growth Factor β (TGFβ) 1. TGFβ is a potent inducer of the epithelial-mesenchymal transition (EMT), which initiates metastasis by favoring cell migration and invasion. However, at metastatic sites, cancer cells have to initiate the reverse process, called mesenchymal-epithelial transition (MET), which requires the inhibition of TGFβ activity 2. Advanced CRCs are usually non-responsive to TGFβ signaling due to mutations either in the TGFβ receptors or in the Mothers Against Decapentaplegic Homolog 4 (SMAD4) mediator 3, 4. In addition, cancer cells use various mechanisms for TGFβ signaling inhibition, including the expression of TGFβ negative regulators, as BMP and Activin Membrane-Bound Inhibitor Homolog (BAMBI) 5. Despite the relevance of TGFβ activity in CRC metastatic progression, the role of other TGFβ inhibitors, such as CD109, has been poorly characterized.

A recent proteomic analysis of secreted proteins in the conditioned media of metastatic CRC cell lines identified an increased abundance of CD109 6, expressed either as a GPI-anchored membrane glycoprotein located in the lipid rafts of the plasma membrane 7 or as a soluble secreted form 8. CD109 belongs to the α2-macroglobulin superfamily, which plays crucial roles in protease inhibition, immune modulation, transport, signaling, and structural functions necessary for maintaining physiological homeostasis and responding to environmental changes 9. CD109 expression was initially reported in endothelial cells, platelets and hematopoietic progenitor cells, where its primary function is still uncertain 8. In cancer, CD109 was reported to be overexpressed in glioblastoma (GBM), lung cancer, squamous cell carcinoma, basal-like breast carcinoma and hepatocellular carcinoma (see 8 for a review).

Cancer-related CD109 functions remain conflicting and cancer cell context-dependent 10. CD109 is involved in the negative regulation of the TGFβ signaling in skin fibrosis, and squamous cell carcinoma cells 8, 10, driving TGFβ receptors degradation in caveolae 11. Furthermore, soluble CD109 hijacks TGFβ apart from the TGFβ receptors in keratinocytes 12, leading to the inhibition of TGFβ signaling. CD109 also promotes the activation of the Signal Transducer and Activator of Transcription 3 (STAT3) pathway in psoriasis 13 and lung cancer metastasis 14, associates with the epidermal growth factor receptor (EGFR) to regulate the AKT/mechanistic Target of Rapamycin (mTOR) signaling pathway in lung cancer 15, and supports tumorigenicity in cervical squamous cell carcinoma via CD109/EGFR/STAT3 signaling 16. CD109 also participates in interleukin 6 (IL-6)-mediated activation of Janus Kinase (JAK)/STAT3 through Interleukin 6 Cytokine Family Signal Transducer (*IL6ST*/GP130) for the maintenance of stemness, tumorigenicity and drug resistance in GBM 17. Despite all these results, CD109-mediated STAT3 activation is controversial, with conflicting findings in lung carcinoma 10, 14 and GBM 14, 18. Moreover, CD109 was reported to associate with latent TGFβ binding protein-1 (LTBP1) to enhance TGF-β activation in the lung tumor stroma 10. As indicated, various CD109 interactors (TGFβ, EGFR, LTBP1 and GP130) have been described in different cancers, supporting a cancer cell context effect. Additional studies are needed to better define the molecular mechanisms by which CD109 influences cancer cell biology, particularly in advanced CRC, signaling regulation capacity and potential interactors. To that end, we have used various cell lines representatives of different phenotypes, stemness characteristics and metastatic capacity. In addition, we have established patient-derived organoids (PDOs) from matched primary and metastatic samples obtained from a patient initially treated with XELOX, a combination of capecitabine and oxaliplatin. Following relapse, the patient received FOLFIRI but subsequently developed liver metastases and ultimately died from disease progression. These clinical observations are consistent with the frequent emergence of chemotherapy resistance in CRC patients 19, supporting the necessity to identify the mechanisms that limit the efficacy of the treatment.

In this report, we found an excellent correlation between CD109 and TGFβ expression in CRC patients. CD109 was highly expressed in the CRIS-B mesenchymal subtype associated with poor prognosis. CD109 silencing led to inhibition of STAT3 activation, which promoted the downregulation of LGR5, Wnt inhibition, and subsequent loss of stemness properties, increasing drug sensitivity. Moreover, we found an association of CD109 with Leucine Rich Repeat Containing 8 VRAC Subunit A (LRRC8A), and other volume regulated anion channel (VRAC) subunits, for the regulation of STAT3 activation and drug resistance, via the A-Kinase Anchoring Protein 12 (AKAP12)/Protein Kinase C Alpha (PKCα) signaling axis. In addition, CD109 was critical for tumor homing and metastatic capacity in mouse models. These findings underscore the role of CD109 as a potential target for controlling STAT3 activity and metastatic spread in CRC.

## Methods

### Cell cultures, siRNAs, plasmids and lentivirus production

KM12SM and KM12L4 cells were obtained from the MD Anderson Cancer Center (Houston, TX, USA), HCT-116 and SW620 cells were from the ECACC (Germany). All cell lines were cultured in DMEM containing 10% FBS (Invitrogen, Carlsbad, CA, USA) and antibiotics at 37 °C in a 5% CO_2_ humidified atmosphere. Cells were authenticated by short tandem repeat analysis and periodically tested for mycoplasma. For transient silencing, siRNAs targeting *CD109* (#1 SASI-HS01_00173897; #2 SASI-HS01_00173898), *LRRC8A* (#1 SASI-HS02_00329027; #2 SASI-HS02_00329028)), *AKAP12* (SASI_HS01_00175942), *LGR5* (SASI-HS01_0019980), or scrambled siRNA (5'-AUUGUAUGCGAUCGCAGACCdTdT-3') were purchased from Merck (Darmstadt, Germany) and transfected in cancer cells using JetPrime Reagent PolyPlus (Illkirch, France), according to manufacturer's instructions. Human *CD109* coding sequence was cloned into pcDNA3.1 vector from the plasmid pENTR-CD109, purchased from DNASU (Tempe, AZ, USA). Empty pcDNA3.1 (Mock) was used as control. Stable silencing was carried out by infection with lentiviral vectors (pLKO.1-puro-CMV-tGFP, Merck) containing two different shRNAs against *CD109* (TRCN0000073649 “shCD109 #49” and TRCN000073650 “shCD109 #50”) or a scrambled (“SCR”) shRNA. Infected cells (GFP^+^) were selected in a FACSAria Fusion sorter (BD Biosciences, Franklin Lakes, NJ, USA). Following 3 rounds of selection, over 90% of the cells were GFP^+^.

### Organoid preparation, culture and maintenance

Samples and data from patients included in this study were provided by the IdiPAZ Biobank (PT23-00028), integrated in the Biobanks and Biomodels ISCIII Platform. PDOs were processed following standard operating procedures with the approval of the Ethics and Scientific Committees. Establishment of tumoral organoid culture (primary tumor and metastasis) was performed as previously described 20. Once they were generated, PDOs were embedded in Matrigel (Corning, New York, USA) and cultured in Advanced DMEM/F12 medium with 15 mM HEPES (StemCell, Vancouver, Canada) diluted 1:1 with IntestiCult OGM Hu Comp A (StemCell) and supplemented with Y-27632, a Rock-Inhibitor (Tocris, Bristol, UK), and Primocin (InvivoGene, San Diego, CA, USA). For passaging, Matrigel was removed by treating with Dispase (Thermo Fisher Scientific, Waltham, MA, USA) for 30 min at 37 ºC. The organoids were scraped and the Matrigel was dissolved with a pipette. The resuspended organoids were washed twice with Advanced DMEM/F12 medium containing 15 mM HEPES supplemented with Glutamax (Invitrogen, Carlsbad, CA, USA) after 4 min centrifugation at 400xg at 4 ºC. The pellet was resuspended in 4mL of PBS containing trypsin and Y-27632 and incubated for 5 min at RT. The organoids were then dissociated into small cell clusters by using a 21G needle previously coated with 1% of BSA in PBS, washed twice with DMEM/F12-HEPES supplemented with Glutamax and Y-27632 and resuspended in Matrigel. The mixture was seeded in drops in preheated 6-well plates. The plate was incubated upside down for 20 min at 37 ºC before adding culture medium. Complete media was refreshed every two days.

### Antibodies and reagents

The antibodies against CD109 (sc-271085) and LRRC8A (sc-517113) used for flow cytometry, and ZEB2 (sc-271984), α-tubulin (sc-5386) and CD133 (sc-35537) used for western blot were purchased from Santa Cruz Biotechnologies (Dallas, TX, USA). Anti-phospho-STAT3 (#9145), anti-phospho-SMAD2 (#3108), anti-STAT3 (#4904), anti-SMAD2/3 (#8585), anti-CD109 (#24765) and anti-LRRC8A (#24979) for immunoprecipitation and western blot were from Cell Signaling (Danvers, MA, USA). Antibodies against CDH17 (24339-1-AP), SNAIL2 (12129-1-AP) and AKAP12 (25199-1-AP) were from Proteintech (Rosemond, IL, USA). Anti-GAPDH (ab8245) and anti-LGR5 (144-10545-20) were acquired from Abcam (Cambridge, UK) and RayBiotech (Norcross, GA, USA), respectively. Antibodies against β-actin (TA811000S) and CDH2 (MAB13881) were from Origene (Rockville, MD, USA) and R&D Systems (Minneapolis, MN, USA), respectively. Anti-PKC antibody (HY-P80430) was purchased from MedChemExpress (Monmouth Junction, NJ, USA).

IL-6, Wnt3a, and TGFβ1 were purchased from Peprotech (Cranbury, NJ, USA). The drugs Carboplatin and Cisplatin, and the PKC inhibitor GO6983 were acquired from TargetMol (Boston, MA, USA); Oxaliplatin, CPT-11 (irinotecan) and 5-Fluorouracil were from Selleckchem (Houston, TX, USA). Stattic and Pyridone-6, Navitoclax and the VRAC blocker, DCPIB were from MedChemExpress, Palbociclib was purchased from APExBIO (Houston, TX, USA).

### Immunofluorescence

For protein colocalization, cells were cultured in Matrigel covered coverslips, fixed with 4 % paraformaldehyde, washed, blocked with 2 % BSA and 1 % goat serum in PBS and incubated with primary antibodies (CD109 #24765 and LRRC8A sc-517113; 10 μg/mL) for 2 h, at room temperature. After washing, cells were incubated with secondary antibodies labelled with Alexa Fluor 488 or Alexa Fluor 568 (diluted 1:1000) and DAPI (Molecular Probes, Eugene, OR, USA). Images were acquired in a LEICA TCS SP8 Confocal microscope (Leica).

### Flow cytometry

Cells were detached with 2 mM EDTA in PBS, and incubated with primary antibodies (10 μg/mL) for 45 min in PBS containing human gamma-globulin (20 μg/mL; Merck). After washing, cells were incubated in the same medium with secondary antibodies labelled with Alexa Fluor 468 (diluted 1:300). Fluorescence was assessed in a Cytoflex-S flow cytometer (Beckman-Coulter, Brea, CA, USA).

### Quantitative PCR

Total RNA was isolated from cellular cultures using TRIzol Reagent (Ambion, Austin, TX, USA) and subjected to retrotranscription using M-MLV reverse transcriptase (Promega, Madison, WI, USA). Amplification of cDNA was performed using FastStart Master Mix (Roche, Basel, Switzerland) with specific probes (Universal Probe Library Set, Roche) in a IQ5 Real-Time PCR thermocycler (Bio-Rad, Hercules, CA, USA). PCR profile was 5 min at 95 °C, 40 step cycles (10 s at 95 °C, 30 s at 57 °C) followed by a melt curve from 57 °C to 95 °C for 380 s. *GAPDH* was amplified as loading control. For semi-quantitative PCR of mouse liver homing assays, the profile was: 5 min at 95 °C, 35 three-step cycles (30 s at 94 °C; 30 s at 57 °C and 30 s at 72 °C) and 5 min at 72 °C. Mouse* actb* was amplified as loading control.

### Cell viability assays

5 x 10^3^ cells were incubated with 1 % FBS in DMEM in 96-well plates in the presence or absence of inhibitors or drugs for 48 h. Then, methyl thiazolyl blue tretazolium bromide (MTT, Merck) at 1 mg/mL was added to the medium for 1.5 h. Optical density at 560 nm was measured as a surrogate for cell viability. For organoids, dissociated organoids (3 × 10^3^ cells) were cultured in 10 μL of Matrigel, on 96-well plates with 100 μL of organoid culture media for 72 h. Then, organoids were incubated with drugs for 96 h, and number of viable cells was determined using CellTiter Blue Cell Viability Assay Kit (Promega).

### Cell adhesion, invasion and migration assays

#### Cell adhesion assays

Cells were serum-starved for 3 h and labeled with BCECF-AM (Molecular Probes). A total of 6 × 10⁴ cells were seeded into 96-well plates previously coated with Matrigel (4 μL/mL; Corning). After 25 min, non-adherent cells were removed by washing, and adherent cells were quantified using a POLARstar Galaxy fluorescence analyzer (BMG Labtech).

#### Cell migration assays

A 1 mm incision was made in a cell monolayer which was incubated in medium containing 0.5% serum. Pictures were taken immediately after the incision and after 16 h. Effective cell migration speed was calculated as the distance (in each flank) covered by the cells in 16 h.

#### Cell invasion assays

A total of 6 × 10⁴ cells were seeded into 8-μm pore-size Transwell inserts (Corning) coated with 35 μL of DMEM containing Matrigel (Corning) at 3 mg/mL. 4% serum in the lower compartment was used as chemoattractant. After 48h, non-invading cells were removed, whereas invading cells were fixed with 4% paraformaldehyde (Sigma-Aldrich) for 10 min, stained with crystal violet (Sigma-Aldrich) for 30 min, and counted under a microscope.

### *In silico* analysis of gene expression databases

Gene expression by stage and prognostic value of genes of interest were analyzed in GSE17538 (n = 244) and GSE39582 (n = 585) colon cancer patient databases. The mRNA expression values of tested genes were normalized by calculating the z-scores prior to statistical analysis. Association with metastasis of gene expression was tested in GSE131418 database (n = 1135), which includes paired samples of metastatic and primary tumors. Association with mutational status and molecular subtypes was carried out using GSE39582. Gene expression correlations were calculated using Pearson correlation coefficients in GSE1433, GSE33113, GSE37892, and GSE44076 databases (for tumor samples) and in GSE59857 database (for colon cancer cell lines). TCGA-Colorectal Adenocarcinoma database was assessed using GEPIA2 web-tool.

### Immunohistochemistry analysis

Human CRC tissues and controls were obtained from the Surgical Pathology Department of the Hospital Clinico San Carlos (Madrid). Immunohistochemistry (IHC) analysis was performed as previously described 6, using the same sample collections and ethical authorizations (Permission #22/550-E).

### Gene expression analyses

For global gene expression analysis, total RNA was isolated from scrambled and CD109-stably KD clones using NucleoSpin RNA kit (Macherey-Nagel, Düren, Germany). RNA quality was assessed in the Agilent 2100 bio-analyzer. Three replicate RNA samples were processed for transcriptomic analysis at the Genomics Unit at the Universidad Complutense de Madrid (Madrid, Spain) using GeneChip® WT PLUS Reagent Kit (Applied Biosystems, Waltham, MA, USA), hybridized with “Clariom™ S Array, human” (Applied Biosystems) and scanned with a “GeneChip® Scanner 3000 7G” (Applied Biosystems). Fold-changes between experimental conditions were calculated as the ratio between the mean of the gene expression signals. Statistical analysis was performed with e-bayes limma included in Transcriptome Analysis Console (Applied Biosystems). Gene Ontology, GSEA, KEGG and Reactome analyses of transcriptomic data were performed by EGO Genomics (Salamanca, Spain). Differentially expressed genes were determined by t-test after ranked bin transformation. Benjaimini-Hochberg procedure was used to calculate the FDR. Transcription regulators predicted to be significantly activated/inhibited were obtained using Ingenuity Pathway Analysis (IPA). Catrin database (Catalogue of Transcriptional Regulatory Interactors) was used to determine the transcription factors that regulate the expression of genes of interest with highest score.

### Western blot, immunoprecipitation and interactomic analyses

KM12SM and SW620 cells were lysed in buffer containing 1% Igepal (NP-40), 100 mM NaCl, 2 mM MgCl₂, 10% glycerol, and 50 mM Tris-HCl, supplemented with protease inhibitors (Roche, Basel, Switzerland) and phosphatase inhibitors (Sigma-Aldrich, St. Louis, MO, USA). One milligram of total cell lysate was incubated for 16 h with Protein G-Sepharose beads (Sigma-Aldrich) and either anti-CD109, anti-LRRC8A or control antibodies (2 μg). Immunoprecipitates were washed with lysis buffer, resuspended in Laemmli sample buffer, and resolved by SDS-PAGE, followed by Western blot analysis. For Western blot analysis, cells were lysed as described above, and 75 μg of total protein were resolved by SDS-PAGE and transferred onto nitrocellulose membranes. Membranes were incubated with the appropriate primary antibodies (1 μg/mL), followed by incubation with horseradish peroxidase (HRP)-conjugated secondary antibodies (diluted 1:2000, Thermo-Fisher Scientific). Protein bands were visualized using SuperSignal™ West Pico Chemiluminescent Substrate (Thermo-Fisher Scientific) and detected with the ChemiDoc™ Imaging System (Bio-Rad). Band quantification was performed using MultiGauge software (Fujifilm, Santa Ana, CA, USA).

For proteomic analysis of co-immunoprecipitated (co-IP) proteins, KM12SM and SW610 cells were lysed and subjected to immunoprecipitation as described above. Co-IP proteins were separated by SDS-PAGE, in-gel digested with trypsin, and the resulting peptides were analyzed by mass spectrometry using a Q-Exactive instrument (Thermo Scientific). Mass spectra data were analyzed with Proteome Discoverer (1.4.0.288) (Thermo Fischer Scientific) and searched against the human Uniprot Database using the Sequest search engine. Proteins were considered positive interactors when they displayed at least a three-fold increase in the number of unique peptides compared with the control immunoprecipitation 21. Analysis of co-IP proteins was carried out as in Gene Expression Analyses section.

### TOP/FOP assay

TOP/FOP reporter assays were performed to evaluate Wnt/β-catenin signaling activity as previously described 22. Briefly, the 8×TOPFlash and 8×FOPFlash luciferase reporter plasmids, kindly provided by Dr. A. Muñoz (IIB-CSIC), were transfected into KM12SM or SW620 cells. Following the indicated treatments, cells were lysed and Firefly and Renilla luciferase activities were measured using the Dual-Luciferase Reporter Assay System (Promega), according to the manufacturer's instructions. In the TOPFlash construct, Firefly luciferase expression is driven by a β-catenin-responsive promoter, whereas the corresponding promoter is mutated in the FOPFlash vector, which therefore serves as a measure of basal luciferase activity. Firefly luciferase activity was normalized to Renilla luciferase activity to account for transfection efficiency, as Renilla luciferase is expressed under the control of a constitutive promoter.

### Patch-clamp experiments

Cells transfected with siRNAs were seeded onto 35-mm plastic Petri dishes previously coated with poly-L-lysine (6 μg/cm²; Sigma-Aldrich) for 1 h at 37 °C. Whole-cell recordings were obtained as previously described 23, using either an Axopatch 200A amplifier (Axon Instruments) or an EPC-10 USB amplifier (HEKA Electronics). Currents were acquired at 33 kHz and filtered at 1 kHz. pClamp 10 (Molecular Devices), PatchMaster, and FitMaster (HEKA Electronics) software were used for pulse generation and data acquisition. Chloride currents (ICl^-^) through VRAC were measured in cells voltage-clamped at 0 mV and subjected every minute to 400-ms voltage pulses from -100 mV to +100 mV in 50-mV increments. For hypotonic shock experiments, pipettes (4-6 MΩ) were filled with an internal solution containing 100 mM N-methyl-D-glucamine chloride (NMDG-Cl), 1.2 mM MgCl_2_, 1 mM EGTA, 10 mM HEPES, 2 mM Na_2_ATP, and 0.5 mM Na_3_GTP (pH 7.3; 300 mOsm/L). The external solution contained 100 mM NMDG-Cl, 0.5 mM MgCl_2_, 5 mM KCl, 1.8 mM CaCl_2_, 5 mM glucose, and 10 mM HEPES (pH 7.32). Osmolarity was adjusted with mannitol to 310 mOsm/L for isotonic conditions and 220 mOsm/L for hypotonic conditions. The magnitude of hypotonicity-induced VRAC activity was determined as the difference between the currents recorded after 10 min of hypotonic perfusion and those measured immediately before perfusion. Whole-cell ICl^-^ currents were also recorded using pipettes (4-6 MΩ) filled with a hypertonic internal solution of low intracellular ionic strength (IS ~ 0.06 M) containing 50 mM CsCl, 1 mM EGTA, 1 mM MgCl_2_, 10 mM HEPES, 2 mM Na_2_ATP, and 0.2 mM Na_3_GTP (pH 7.25; 412 mOsm/L, adjusted with mannitol). In these experiments, the external isotonic solution contained 100 mM NaCl, 6 mM KCl, 1 mM MgCl_2_, 2 mM CaCl_2_, 10 mM glucose, and 10 mM HEPES pH 7.3; 310 mOsm/L, adjusted with mannitol. ICl^-^ currents were evaluated 2 min after establishment of the whole-cell configuration, a time point at which substantial VRAC activity was already detectable in cells transfected with control (scrambled) siRNA.

### Senescence assays

KM12SM shCD109, KM12SM SCR, SW620 SCR, and SW620 shCD109 cell lines were treated with 5 µM Palbociclib for 7 days, changing the treatment every 48 h, with the last change occurring at 72 h. For β-gal staining, cells were fixed with 0.5% glutaraldehyde in PBS for 15 min at room temperature. The fixed cells were then washed three times with 1mM MgCl_2_ pH:6.0. Then, an X-Gal staining solution was added (1mg/mL N-N-Dimethyl-formamide, 100 mM K_3_[Fe(CN)_6_], and 100 mM K_4_[Fe(CN)_6_]·3H_2_O) in PBS/MgCl_2_. The X-Gal solution was added to the cells and incubated overnight at 37°C. Quantification was performed using ImageJ and data are represented as the number of cells staining positive for β-Gal normalized to total cell number.

### *In vivo* experiments

The Ethical Committees of the CSIC and Community of Madrid approved the protocols used for the experimental work with animals (PROEX 190.1/20). For xenograft tumor growth, 5 x 10^6^ SW620 cells were inoculated subcutaneously in PBS 0.1% glucose into NGS mice in both flanks (n = 3). In all cases, we followed the standard calculation of sample size in animal studies, employing the formula n = 2σ^2^·(Z^α/2^+Z^β^)^2^/d^2^ to calculate the number of mice for the assays. Tumor volumes were measured twice per week for 6 weeks. For liver homing assessment, NSG mice (n = 3) were inoculated in spleen with 1x10^6^ KM12SM transfectants in 0.1 mL PBS, and euthanized 96 h after inoculation. RNA was isolated from liver using TRIzol (Thermo Fisher Scientific) and analyzed by semiquantitative PCR or qPCR to amplify human *GAPDH* and, as loading control, murine *Actb*. For liver metastasis, NSG or Swiss Nude mice (at least n = 6 per condition) were inoculated in the spleen with 1x10^6^ KM12SM siRNA or shRNA transfectants and, after 24 h, subjected to splenectomy to avoid local tumor growth. When signs of illness were detected, mice were euthanized and inspected for metastasis in the liver.

### Statistical analyses

All assays were carried out in triplicate. Histograms showed the average value, with the standard deviation as error bars. Gene expression correlations were calculated by using the Pearson correlation p-value. Kaplan-Meier survival analyses were assessed by the log-rank test. All other data were tested for Gaussian distribution using the Kolmogorov Smirnov method and for differences in standard deviations with F test. Assays with two conditions were analyzed by Student *t*-test one-way), whereas those with more than two conditions were analyzed by one-way ANOVA followed by Tukey-Kramer (or Dunnett's for Patch-clamp assays) multiple comparison test post-hoc analysis. In all statistical analyses, the minimum acceptable level of significance was p < 0.05.

## Results

### CD109 expression associates with aggressive mesenchymal subtypes and poor prognosis in colorectal cancer

**Figure 1:**
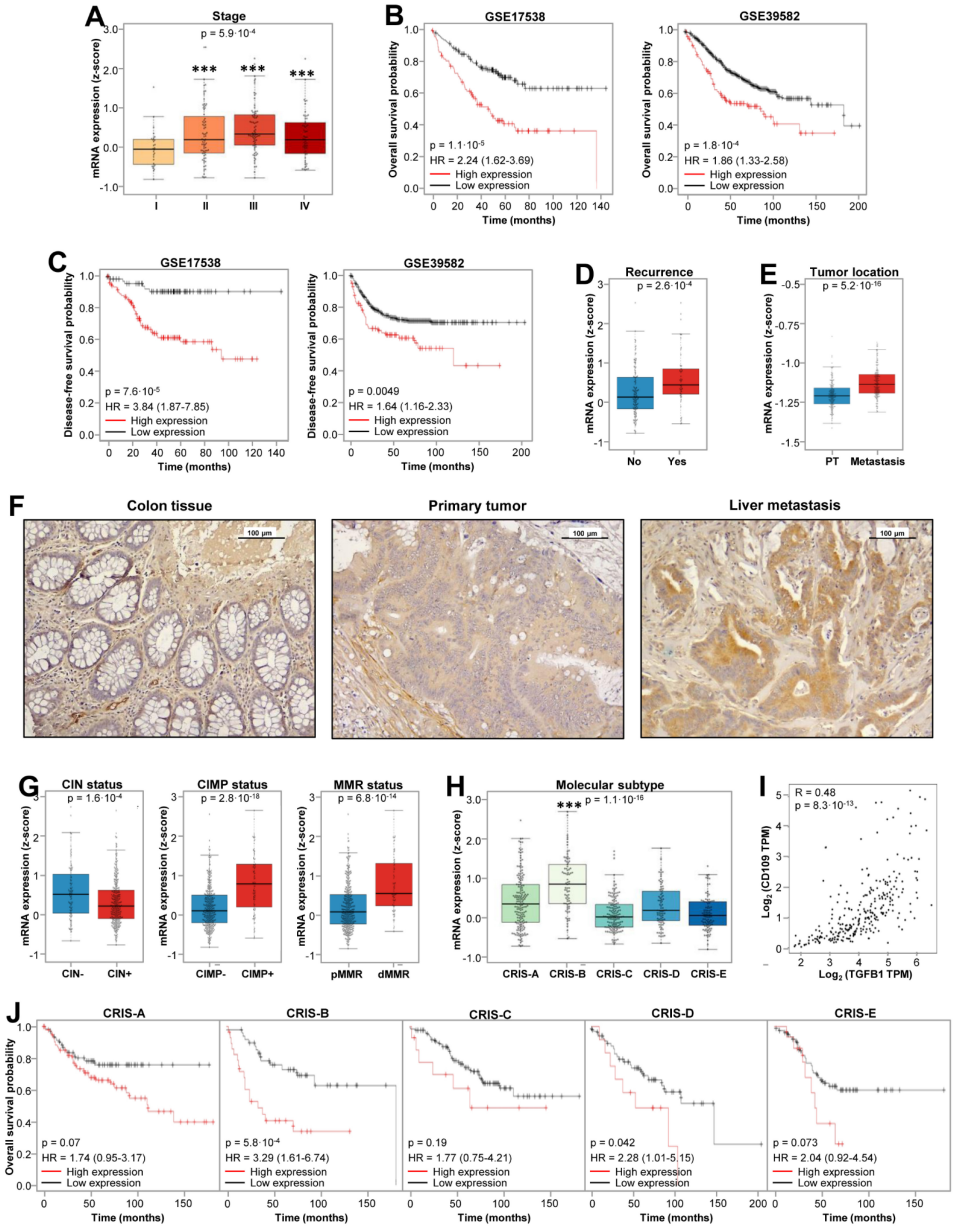
** CD109 associates with poor prognosis in CRC patients.** (A) *CD109* is highly expressed in stages II, III and IV respect to stage I in GSE17538 cohort. ANOVA p-value is indicated. (B) Kaplan-Meier overall survival and (C) and disease-free survival analysis of CRC patients in the indicated databases according to *CD109* expression. Log-rank test p-values and Cox regression model HR are indicated. (D) *CD10*9 expression according to recurrence status in GSE17538 cohort, and (E) *CD109* expression in paired primary tumor and liver metastasis in GSE131418 cohort. *T* test p values are shown inside each box plot. (F) Representative images of CD109 expression in healthy colon, primary tumor and liver metastasis by immunohistochemical analyses. (G) *CD109* expression according to CIN CIMP and MMR status in GSE39582 cohort. (H) *CD109* expression (z-score) distribution according to the CRIS classifiers in GSE39582 database. CD109 expression was significantly increased in CRIS-B, compared to other subtypes (***, p < 0.001). (I) Correlation analysis of the expression levels of *CD109* and *TGFB1* in TCGA colon cancer cohort using GEPIA webpage. Pearson correlation coefficient and p-value are shown inside the panel. TPM: transcripts per million (J) Kaplan-Meier overall survival analyses by CRIS subtype in the GSE39582 cohort according to *CD109* expression. Log-rank test p-values and Cox regression model HRs (Hazard Ratios) are indicated.

To address its clinical relevance, CD109 expression was investigated in different CRC patient datasets and tissue samples. CD109 was overexpressed in stages II, III and IV respect to stage I (**Fig. 1A**), showing association with lower overall- and disease-free survival (**Fig. 1B, 1C**), patient recurrence (**Fig. 1D**) and higher expression in metastasis respect to primary tumors (**Fig. 1E**). The increased expression of CD109 in metastatic tissues compared with primary tumors and normal colon mucosa was confirmed by IHC staining (**Fig. 1F**). Furthermore, CD109 expression revealed a negative correlation with chromosomal instability (CIN), but a positive association with CpG-island methylator phenotype (CIMP) and deficient mismatch repair (MMR) status (**Fig. 1G**), which also associate with poor prognosis. Regarding CRC molecular classification 24, highest CD109 expression was associated with the mesenchymal Colorectal Cancer Intrinsic Subtype B (CRIS-B) subtype (**Fig. 1H**), characterized by high inflammation, EMT, and TGFβ activity 24. Indeed, CD109 showed an excellent correlation with TGFβ1 expression (R = 0.48), according to GEPIA 25 (**Fig. 1I**). High CD109 expression in patients also correlated with a poor outcome in CRIS-B and CRIS-D (**Fig. 1J**). In summary, high CD109 expression is significantly associated with CRC advanced stages, mesenchymal subtypes, TGFβ expression and shorter survival of patients.

### Loss of CD109 inhibits STAT3 activation, IL6 expression and cell proliferation

In CRC, STAT3 signaling promotes cell proliferation, migration and invasion 26, 27, and has been associated with poor prognosis 28. We explored the effect of knocking down (KD) CD109 on STAT3 activation in four CRC metastatic cell lines: SW620, KM12SM, KM12L4 and HCT-116. SiRNA KD cell lines showed a significant decrease of pSTAT3 (Y705) activation, except HCT-116 (**Fig. S1A**). Validation of CD109 KD was also tested by qPCR (**Fig. S1B**). Furthermore, we assessed the expression of several STAT3 transcriptional targets following CD109 silencing by qPCR. Our results showed a substantial decrease in well-established targets as IL6, MCL1, SOCS3 and BCL2L1 after CD109 silencing, with particularly strong repression of IL6 in both cell lines (**Fig. S1C**). To exclude potential off target effects of the CD109 siRNA on STAT3 activation, CD109-silenced cells were transfected with a vector overexpressing CD109 (OE CD109). Restoration of CD109 expression rescued phospho-STAT3 levels (**Fig. S1D**). Moreover, CD109 overexpression increased the activation of STAT3, confirming the role of CD109 in regulating STAT3 activation in CRC cells.

Based on these results, we selected KM12SM and SW620 cells for the remaining studies. Whereas CD109 silencing have no effect in cell adhesion, migration or invasion capacities in both cell lines (**Fig. S1E, F, G**), we observed a clear decrease of cell proliferation in KM12SM (**Fig. S1H**). To further validate CD109 effects on cell proliferation, we carried out stable CD109 silencing using two specific shRNAs and scrambled shRNA as a control. Silencing efficiency and specificity were validated by Western blotting, qPCR and flow cytometry in both cell lines (**Fig. S2A, B, C**), being shRNA#49 more efficient than shRNA#50. Cell proliferation was clearly diminished in KM12SM cells, while SW620 cells growth remained unaffected in cell culture (**Fig. S2D**). To clarify this discrepancy, we carried out *in vivo* xenograft experiments that showed a clear decrease of tumor growth after subcutaneous inoculation of SW620 KD cells (**Fig. S2E**), supporting anti-proliferative effects of CD109 silencing *in vivo*. To corroborate these effects on STAT3 activation and cell proliferation, we investigated the use of Stattic, a selective STAT3 inhibitor 29, and Pyridone-6, a pan-JAK/STAT inhibitor 14, which were highly effective in reducing STAT3 activation in both cell lines (**Fig. S3A**). Interestingly, CD109-silenced cells were more sensitive than scrambled cells to 1-2 µM Stattic (**Fig. S3B**), and to 0.1-1 µM Pyridone-6 (**Fig. S3C**). Collectively, these results indicate that CD109 depletion inhibits STAT3 activation and, subsequently, IL6 expression in metastatic CRC, reduces proliferation and increases cell sensitivity to STAT3 inhibitors.

### Loss of CD109 associates with Wnt inhibition, increased inflammation-associated pathways, and cellular senescence

**Figure 2:**
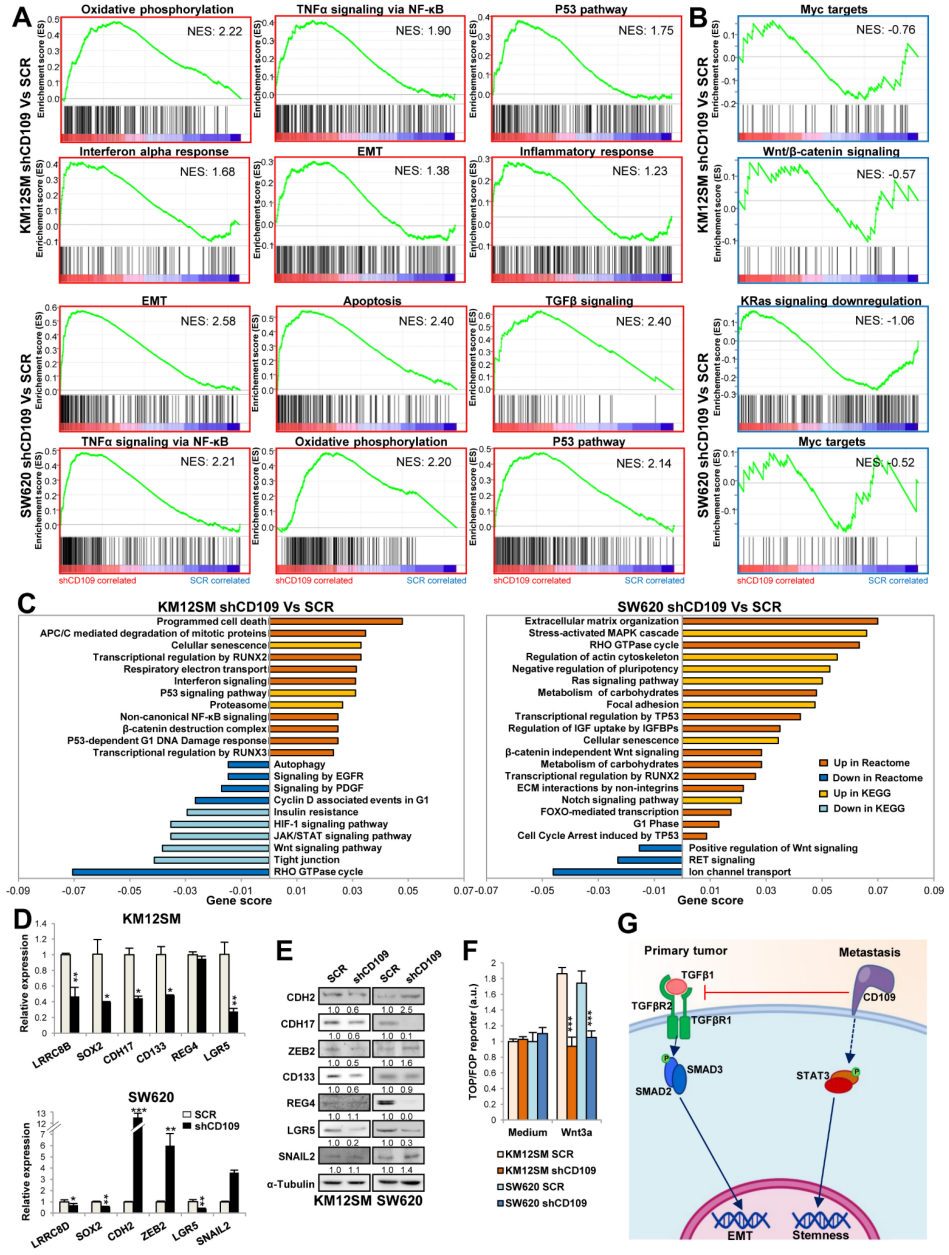
** Transcriptomic analyses of CD109-silenced cells**. (A, B) Gene set enrichment analysis (GSEA) using hallmark gene sets from the Molecular Signature Database (MSigDB) was performed on genes upregulated (A) or downregulated (B) in *CD109*-silenced cells (shCD109). Normalized Enrichment Scores (NES) are shown inside each panel. (C) Pathways significantly dysregulated in shCD109 stable transductants according to KEGG and Reactome analyses. (D) Quantitative PCR of the indicated genes in SCR and shCD109 stable transductants (*, p <0.05; **, p < 0.01; ***, p < 0.001) (E) Western blot analysis of the indicated proteins in the same transductants as in A. Band quantification is shown below each lane. (F) TOP/FOP 24h assays were done in the indicated lines after treatment with Wnt3a (500 ng/mL). The ratio TOP/FOP was significantly decreased in treated cells after *CD109* silencing (***, p < 0.001). Data are representative of three independent experiments. (G) Schematic representation of TGFβ and CD109 functional activities. In primary tumors, TGFβ activity promotes SMAD activation and EMT, whereas CD109 inhibits TGFβ-triggered signaling. In metastatic tumors, where TGFβ signaling is commonly abolished, CD109 promotes STAT3 activation, Wnt signaling and stemness maintenance.

To further understand these findings, we investigated global gene expression alterations in CD109 KD compared with scrambled (SCR) cells. Altered genes were filtered based on a fold-change ≥ 2 and a false discovery rate (FDR) ≤ 0.05 in a triplicate analysis. GSEA hallmarks analysis revealed some commonalities in both cell lines after CD109 silencing, such as the increase in inflammation-associated pathways, oxidative phosphorylation (OXPHOS), Tumor Necrosis Factor alpha (TNFα) signaling via Nuclear Factor of Kappa Light Polypeptide Gene Enhancer in B-Cells Inhibitor (NF-κB), P53 pathway, and EMT (**Fig. 2A**), together with a down-regulation of MYC targets (**Fig. 2B**). Beyond these similarities, KEGG and REACTOME analyses revealed distinct cell line-dependent alterations. In KM12SM, we noticed a preferential downregulation of JAK/STAT, HIF-1, EGFR, and PDGF signaling, which was consistent with a strong reduction in proliferation, STAT3 activation and IL6 expression (**Fig. 2C**). In contrast, SW620 cells showed “suppression of positive regulation of Wnt signaling”, “RET signaling” and “ion channel transport”. Upregulated pathways also diverged: whereas KM12SM cells showed enrichment in “programmed cell death”, and “β-catenin destruction complex”, SW620 cells showed activation of Mitogen-Activated Protein Kinase (MAPK) (C-Jun N-Terminal Kinase 1 (JNK)/p38), Rho-GTPase cycle, Notch signaling, and FOXO-mediated transcription.

At the single-gene level, *CD109* silencing consistently suppressed pluripotency markers (SRY-Box Transcription Factor 2 (*SOX2*)*, NANOG*) and the intestinal stem cell marker Leucine Rich Repeat Containing G Protein-Coupled Receptor 5 (*LGR5*) in both cell lines, with additional downregulation of *CD133* in KM12SM (**Table S1**). These alterations, together with Dickkopf Wnt Signaling Pathway Inhibitor 1 (*DKK1*) upregulation in SW620 would support reduced Wnt signaling. The observed enrichment in EMT was associated either with an increased expression of mesenchymal markers (cadherin 2 (*CDH2*), fibronectin 1 (*FN1*), vimentin (*VIM*), snail2 (*SNAI2*) and Zinc Finger E-Box Binding Homeobox 2 (*ZEB2*)) in SW620, or with the loss of epithelial markers (keratin 8 (*KRT8*), Zona Occludens 1 (*ZO-1*), E-cadherin and cadherin 17 (*CDH17*)) in KM12SM. Additionally, we observed a senescence signature where KM12SM showed an upregulation of p16^INK4A^ (*CDKN2A*), C-C Motif Chemokine Ligand 20 (*CCL20*), and Forkhead Box O1 (*FOXO1*), while SW620 displayed strong induction of p21^CIP1^ (*CDKN1A*) and Cyclin D2 (*CCND2*) (**Table S1**). The increase in senescence was confirmed by β-galactosidase staining after treatment with palbociclib, a senescence-promoter agent. CD109 KD cells showed stronger staining when compared with SCR cells (**Fig. S4**). Gene expression alterations were confirmed by qPCR (**Fig. 2D**), and correlated with alterations in protein abundance by Western blot (**Fig. 2E**). To confirm the alterations in Wnt signaling, we carried out TOP/FOP assays, which indicated a significant loss of Wnt/β-catenin activity in both cell lines after CD109 silencing (**Fig. 2F**). Collectively, these results suggest that CD109-mediated STAT3 activation regulates stemness and cell proliferation capacities through the regulation of *LGR5* and Wnt signaling in CRC metastatic cells (**Fig. 2G**).

### CD109 associates with LRRC8A and other VRAC subunits

**Figure 3:**
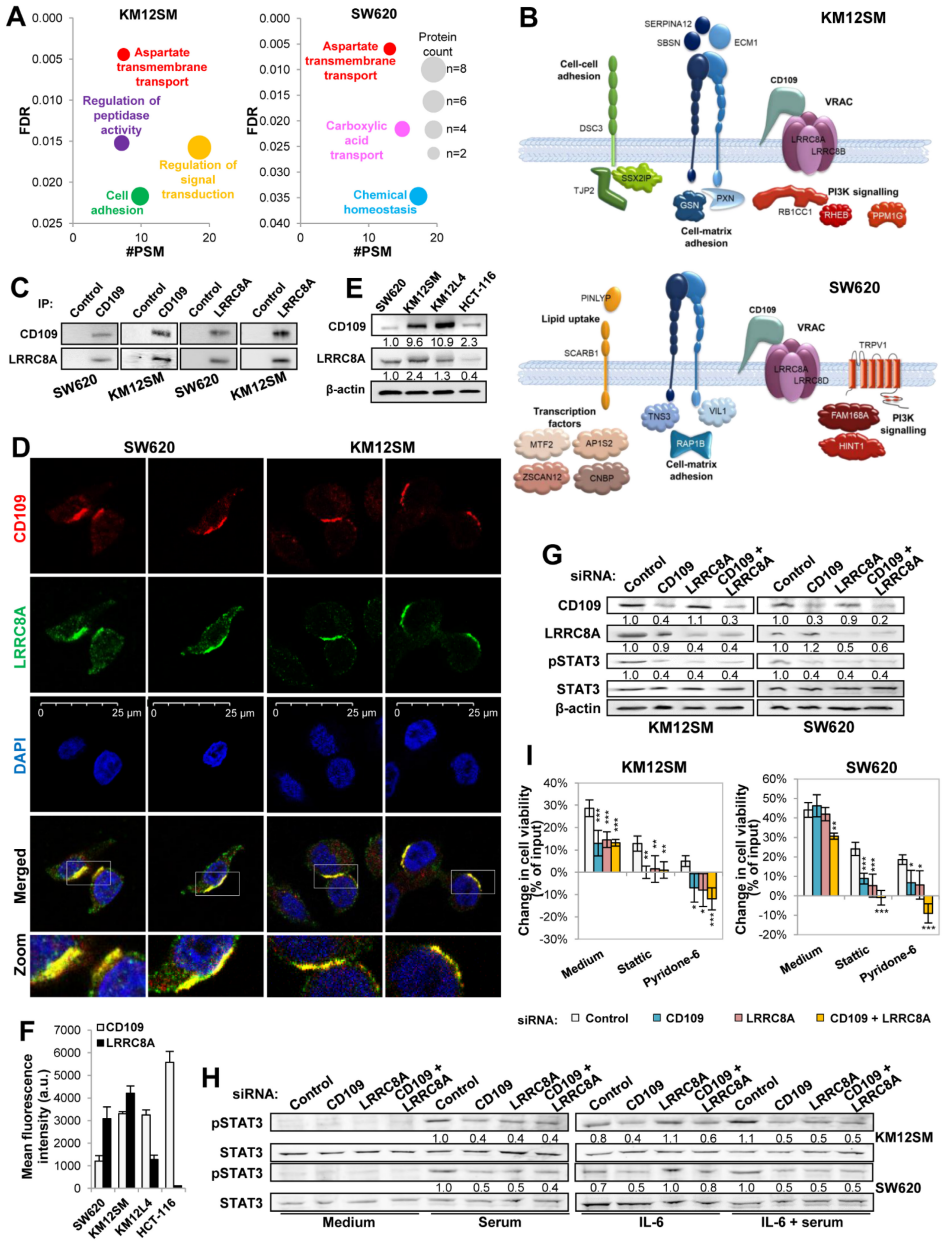
** CD109 associates with LRRC8A.** (A) Gene Ontology Biological Processes of CD109 co-immunoprecipitated proteins selected according to FDR and number of peptide spectral matches. (B) Representative scheme of CD109 co-immunoprecipitated proteins detected by mass spectrometry. (C) Western blot analyses after co-immunoprecipitation assays using anti-CD109, anti-LRRC8A or control antibodies in protein extracts from the indicated cell lines. (D) Confocal microscopy images of the indicated cell lines stained with CD109 and LRRC8A antibodies. (E) Western blot analyses of CD109 and LRRC8A in the indicated cell lines. Band quantification is shown below each lane. (F) Flow cytometry analysis of CD109 and LRRC8A cell surface expression in the indicated cell lines. (G) Effect on STAT3 activation in KM12SM and SW620 cells transfected with control, *CD109*- and/or *LRRC8A*-targeting siRNAs. Band quantification is shown below each band. (H) The same transfectants as in G were kept in starving, exposed to IL-6 (10 ng/mL) and/or serum for 15 min and lysed. The protein extracts were analyzed by Western blot to detect the indicated proteins. The ratios between phosphorylated and total STAT3 are shown between the lanes. (I) Cell viability assays in the presence or absence of Stattic (2 μM) or Pyridone-6 (0.2 μM for KM12SM and 1 μM for SW620 cells) were carried out for 48h. Cell viability was significantly decreased by the silencing of *CD109* and/or *LRRC8A* (*, p < 0.05; **, p < 0.01; ***, p < 0.001). Overall, data are representative of three independent experiments.

To further understand the mechanisms of STAT3 activation by CD109, a protein-protein interaction analysis was carried out in both cell lines using immunoprecipitation (IP) and mass spectrometry (**Table S2**). GO analysis of the interacting proteins revealed a major association of CD109 with “aspartate transmembrane transport” proteins in KM12SM and SW620 cells (**Fig. 3A**). Among the aspartate transmembrane transport proteins, LRRC8A a subunit of the volume-regulated anion channels (VRAC) 30 was selected for further studies, as it was identified in both cell lines (**Table S2, Fig. 3B**). Other interacting proteins, Suprabasin (SBSN) and Extracellular Matrix Protein 1 (ECM1) in KM12SM, and Ras-Related Protein Rap-1b (RAP1B) and Villin 1 (VIL1) in SW620, were involved in cell-cell adhesion, cell-matrix adhesion and PI3K signaling, see a scheme in **Fig. 3B**. The association with LRRC8A (a.k.a. SWELL1) was validated by reciprocal co-IP experiments with CD109 in both cell lines (**Fig. 3C**) and confocal microscopy, which indicated a preferential localization in cell-cell contact areas (**Fig. 3D**). We observed a preferential association of CD109/LRRC8A with LRRC8B in KM12SM cells and with LRRC8D in SW620 (**Table S2**), which was consistent with the downregulation of LRRC8B in KM12SM and LRRC8D in SW620 KD cells after CD109 silencing (**Table S1, Fig. 2D**). CD109 and LRRC8A protein expression was higher in KM12SM and KM12L4 than in SW620 and HCT-116 cells (**Fig. 3E**). Flow cytometry indicated that CD109 was mainly cell membrane-associated in HCT116, in contrast to LRRC8A, which was barely detectable in cell membrane (**Fig. 3F**).

CD109 silencing showed no effect on LRRC8A expression levels; likewise, LRRC8A KD similarly failed to alter CD109 expression using two different siRNAs (**Fig. 3G, Fig. S5A, B**). However, both individual and combined silencing of these proteins consistently inhibited STAT3 phosphorylation, (**Fig. 3G, Fig. S5A**). Given the established role of CD109 as a negative regulator of TGFβ signaling 11, 31, 32, we examined the effects of single and combined CD109 and LRRC8A silencing on SMAD2 activation (**Fig. S6**). In KM12SM cells, no SMAD2 activation was detected, likely reflecting alterations in TGFβ receptor signaling in metastatic cells 1. In SW620 cells, TGFβ-induced SMAD2 phosphorylation was observed, but remained unaffected by CD109/LRRC8A silencing, consistent with the presence of downstream SMAD4 mutations 33 (**Fig. S6**). Together, these findings support the notion that the canonical TGFβ pathway is already largely inactive in these metastatic CRC models. In contrast, according our transcriptomic data, CD109-mediated effects in metastatic CRC cells may be primarily driven through non-canonical, SMAD-independent TGFβ signaling pathways, including MAPK, PI3K/AKT, Rho GTPases, and NF-κB signaling.

### CD109 knockdown reduces VRAC activity in metastatic CRC cells under hypotonic and low intracellular ionic strength conditions

Whole-cell patch-clamp recordings were used to assess the role of CD109 in VRAC activity in metastatic CRC cells. Hypotonic stimulation induced robust VRAC-like chloride currents in control cells (**Fig. S7A, C**), which were larger in SW620 cells (**Fig. S7D**). These currents were markedly inhibited by the LRRC8A blocker DCPIB, supporting their identification as VRAC-mediated currents. In KM12SM cells, CD109 knockdown significantly reduced these currents, to a similar extent as LRRC8A silencing (**Fig. S7A, B**), whereas in SW620 cells, although both CD109 and LRRC8A knockdown decreased the amplitude of hypotonicity-induced currents, such reduction did not reach statistical significance for LRRC8A-targeting siRNA, most likely because of the high variability of the response (**Fig. S7C, D**). We next examined VRAC activation induced by intracellular dialysis with a hypertonic solution of low ionic strength, a well-established stimulus that activates VRAC independently of extracellular hypotonic exposure 34, 35 (**Fig. S8**). In KM12SM cells, knockdown of either CD109 or LRRC8A significantly reduced current density compared with cells transfected with control siRNA (**Fig. S8A, B**). A similar effect was observed in SW620 cells, in which knockdown of either CD109 or LRRC8A also significantly decreased low-ionic-strength-induced chloride currents, with LRRC8A silencing producing the strongest inhibition (**Fig. S8C, D**). Taken together, these findings suggest that CD109 positively regulates VRAC activity in metastatic CRC cells. The observation that CD109 knockdown attenuated VRAC activation under both hypotonic and low intracellular ionic strength conditions supports a role for CD109 in the efficient activation and/or maintenance of LRRC8A-containing VRAC mediated currents. This is consistent with a functional association between CD109, the LRRC8A channel subunit, and VRAC activity in these metastatic CRC cell models.

### The CD109/LRRC8A complex regulates IL-6-mediated STAT3 activation

Previously, CD109 was reported to interact with GP130 to promote activation of the IL6/STAT3 pathway in GBM 17. To further investigate the relationship between CD109/LRRC8A and STAT3 signaling, we examined STAT3 activation after IL-6 stimulation in cells depleted of CD109 or LRRC8A. Silencing of either protein using two independent siRNAs prevented STAT3 activation under serum conditions (**Fig. 3H, Fig. S5D**). Consistently, IL-6 failed to induce STAT3 phosphorylation in CD109-silenced cells, regardless of whether cells were stimulated with serum, IL-6, or the combination of both (**Fig. 3H, Fig. S5D**). In contrast, LRRC8A silencing led to increased STAT3 activation by IL-6, suggesting that LRRC8A depletion increases CD109 availability for interaction with the IL-6 receptor GP130. Supporting this model, simultaneous silencing of CD109 and LRRC8A restored pSTAT3 levels to baseline (F**ig. 3H, Fig. S5C**). Consistent with these observations, LRRC8A KD cells showed a similar sensitivity to Stattic and Pyridone-6 comparable to that observed in CD109 silenced cells (**Fig. 3I, Fig. S5D**). Double silencing CD109/LLRC8A was more effective in both cases, particularly for Pyridone-6. Collectively, these findings support a model in which the CD109/LRRC8A complex regulates IL-6-mediated STAT3 activation by modulating CD109 accessibility to GP130.

### AKAP12 associates with the CD109/LRRC8A complex and correlates with poor prognosis in CRC

**Figure 4:**
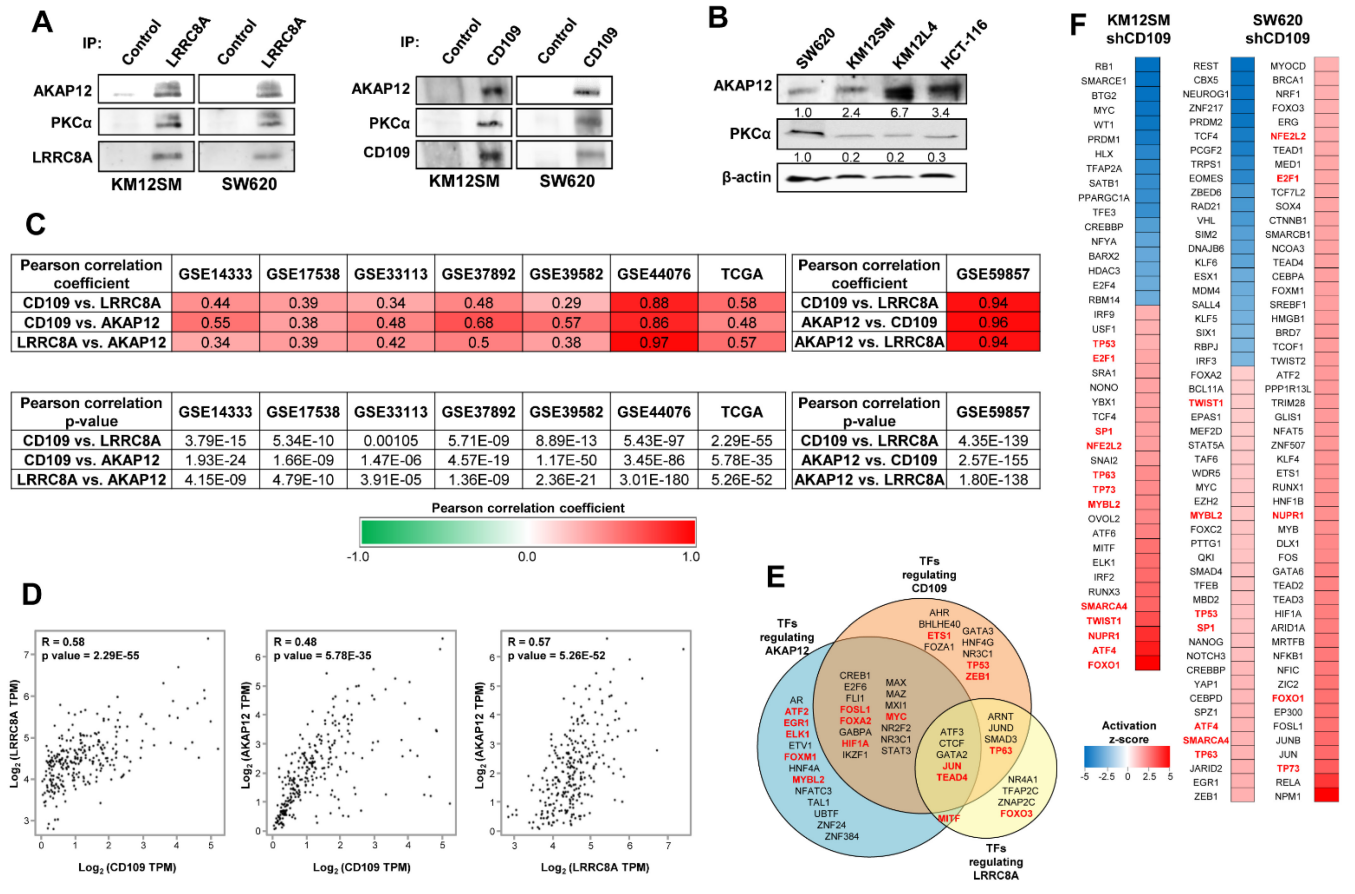
** Association and correlation of CD109, LRRC8A and AKAP12 expression levels in colorectal cancer.** (A) Western blot analysis after LRRC8A, CD109 or control IPs to detect PKCα and AKAP12 in the indicated cell lines. (B) Western blot analysis of PKCα and AKAP12 in CRC cell lines. (C) Pearson correlation coefficient and p-value of the expression levels of the indicated pairs of genes in the indicated CRC patient cohorts and cell line database (GSE59857). (D) Correlations between the expression levels the indicated genes in TCGA CRC cohort. Pearson correlation coefficient and p-value are shown inside each panel. (E) Transcription factors that regulate the expression of the indicated genes according to Catrin database. In red, those found in (F). (F) Transcriptional regulators whose activation z-scores are significantly altered after CD109 silencing according to the Ingenuity Pathway Analysis. In red those found in both cell lines.

To explore the molecular links between the CD109/LRRC8A complex and STAT3 activation, we searched for potential LRRC8A protein mediators. Previous studies have reported that LRRC8A interacts with AKAP12 36, 37 and promotes hepatocellular carcinoma metastasis through PKCα signaling 38, a known STAT3 activator. Indeed, AKAP12 functions as a scaffold protein anchoring PKC proteins to the plasma membrane 39. We confirmed the association of LRRC8A and CD109 with AKAP12 and PKCα in CRC cells by performing two IPs for LRRC8A and CD109 followed by Western blot (**Fig. 4A**). These experiments support the formation of a multiprotein complex comprising CD109, LRRC8A, AKAP12 and PKCα. We then examined the expression of AKAP12 and PKCα in metastatic CRC cell lines. Highly metastatic cell lines exhibited elevated AKAP12 expression and variable PKCα levels (**Fig. 4B**).

From a clinical perspective, *CD109*, *LRRC8A*, and *AKAP12* expression levels showed strong correlation across multiple CRC patient datasets (**Fig. 4C, D**). These genes also shared a large set of upstream transcription factors, suggesting coordinated transcriptional regulation (**Fig. 4E**). Various of these transcriptional regulators were predicted to be activated after CD109 silencing in both cell lines (**Fig. 4F**). Consistently, *LRRC8A* and *AKAP12* showed expression patterns similar to *CD109*, with significant enrichment in the aggressive CRIS-B subtype (**Fig. S9A**). Moreover, high expression of *LRRC8A* and *AKAP12* was consistently associated with poor clinical outcomes (**Fig. S9B**), particularly the mesenchymal CRIS-B subtype (**Fig. S9C, D**). *LRRC8A*, in particular, displayed strong hazard ratios and statistical significance in CRIS-B patients. Collectively, these results support the association of AKAP12/PKCα with the complex and highlight a strong co-expression pattern among *CD109*, *LRRC8A*, and *AKAP12*, suggesting that their combined expression may serve as a prognostic marker panel in CRC.

### STAT3 inhibition drives LGR5 downregulation and Wnt signaling

**Figure 5:**
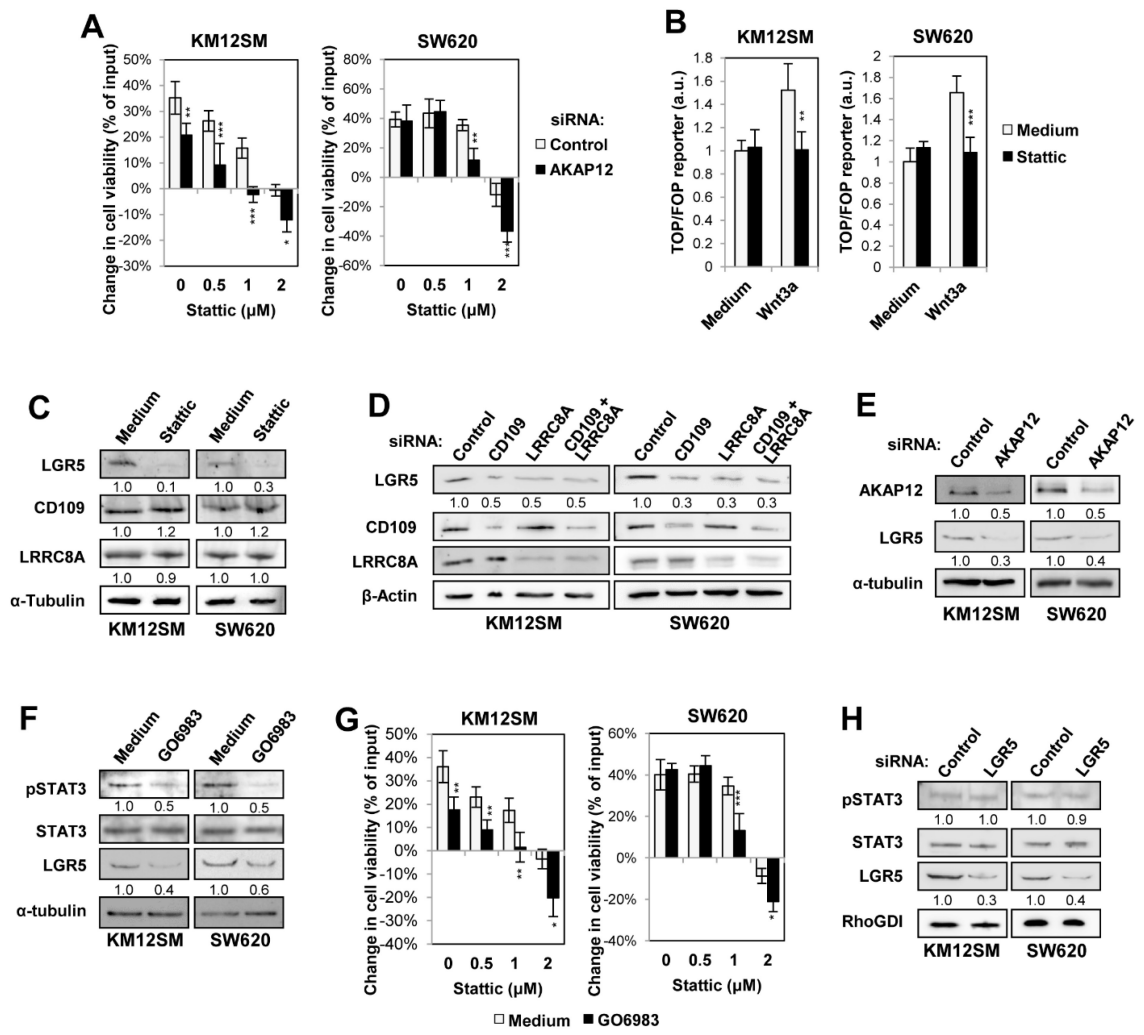
** The axis CD109/LRRC8A/AKAP12 regulates LGR5 expression via STAT3 activation.** (A) KM12SM and SW620 cells were transfected with control or *AKAP12*-targeting siRNAs and subjected to cell viability assays for 48h after Stattic treatment. (B) TOP/FOP assays 24h after treatment with Stattic (1 μM) and/or Wnt3a (500 ng/mL). The ratio TOP/FOP was significantly decreased in Stattic-treated cells (**, p < 0.01; ***, p < 0.001). (C) LGR5, CD109 and LRRC8A expression levels were analyzed by Western blot following Stattic treatment (1 µM) for 48h. Band quantification is shown below each band. (D) The indicated cell lines were transfected with control, *CD109* and/or *LRRC8A* targeting siRNAs and the cell extracts were analyzed by Western blot to detect the expression of the indicated proteins. (E) The same transfectants as in A were lysed and the extracts analyzed by Western blot to detect AKAP12 and LGR5. (F) STAT3 activation and LGR5 expression in cell lines treated with or without GO6983 (0.3 μM) for 48h. (G) The indicated cells were subjected to cell viability assays in the presence or absence of GO6983 and Stattic. Cell viability was significantly reduced after GO6983 treatment for 48h or AKAP12 silencing (*, p < 0.05; **, p < 0.01; ***, p < 0.001). (H) STAT3 activation in control or *LGR5*-silenced cells. Overall, data are representative of three independent experiments.

We next examined how AKAP12 in combination with the CD109/LRRC8A complex drives STAT3 activation and regulates LGR5 expression in metastatic CRC cells. *AKAP12* silencing increased sensitivity to Stattic, similarly to *CD109* or *LRRC8A* knockdown (**Fig. 5A**). Consistently, Stattic treatment significantly suppressed Wnt signaling activity in both cell lines, as determined by TOP/FOP luciferase assays (**Fig. 5B**), and markedly reduced LGR5 expression, without affecting CD109 or LRRC8A levels (**Fig. 5C**), Moreover, depletion of cursiva: CD109, LRRC8A, or *AKAP12*, either individually or in combination, led to comparable reductions in LGR5 expression and cell viability in KM12SM cells (**Fig. 5D, E, Fig. S10A, B**).

To assess the involvement of PKCα in this regulatory mechanism, cells were treated with the PKCα inhibitor GO6983. PKC inhibition recapitulated the effects of AKAP12 silencing by reducing STAT3 activation, decreasing LGR5 expression (**Fig. 5F**), and impaired cell viability (**Fig. 5G**). Likewise, PKCα inhibition alone or in combination with CD109 silencing caused similar results (**Fig. S10C, D**), suggesting that CD109, LRRC8A, AKAP12, and PKCα form part of the same signaling pathway. In contrast, *LGR5* silencing did not affect STAT3 activation (**Fig. 5H**) supporting a model in which STAT3 functions upstream of LGR5 and Wnt signaling**.** This model is consistent with previous reports showing that the IKKα-STAT3 complex directly binds to and regulates the LGR5 promoter 40. Collectively, these findings indicate that the AKAP12/PKCα axis, downstream of the CD109/LRRC8A complex, regulates STAT3 activity, which in turn controls LGR5-dependent Wnt signaling to sustain cancer stem cell (CSC) properties.

### The CD109/LRRC8A complex confers drug resistance in colorectal cancer and is upregulated in metastatic human organoids

**Figure 6:**
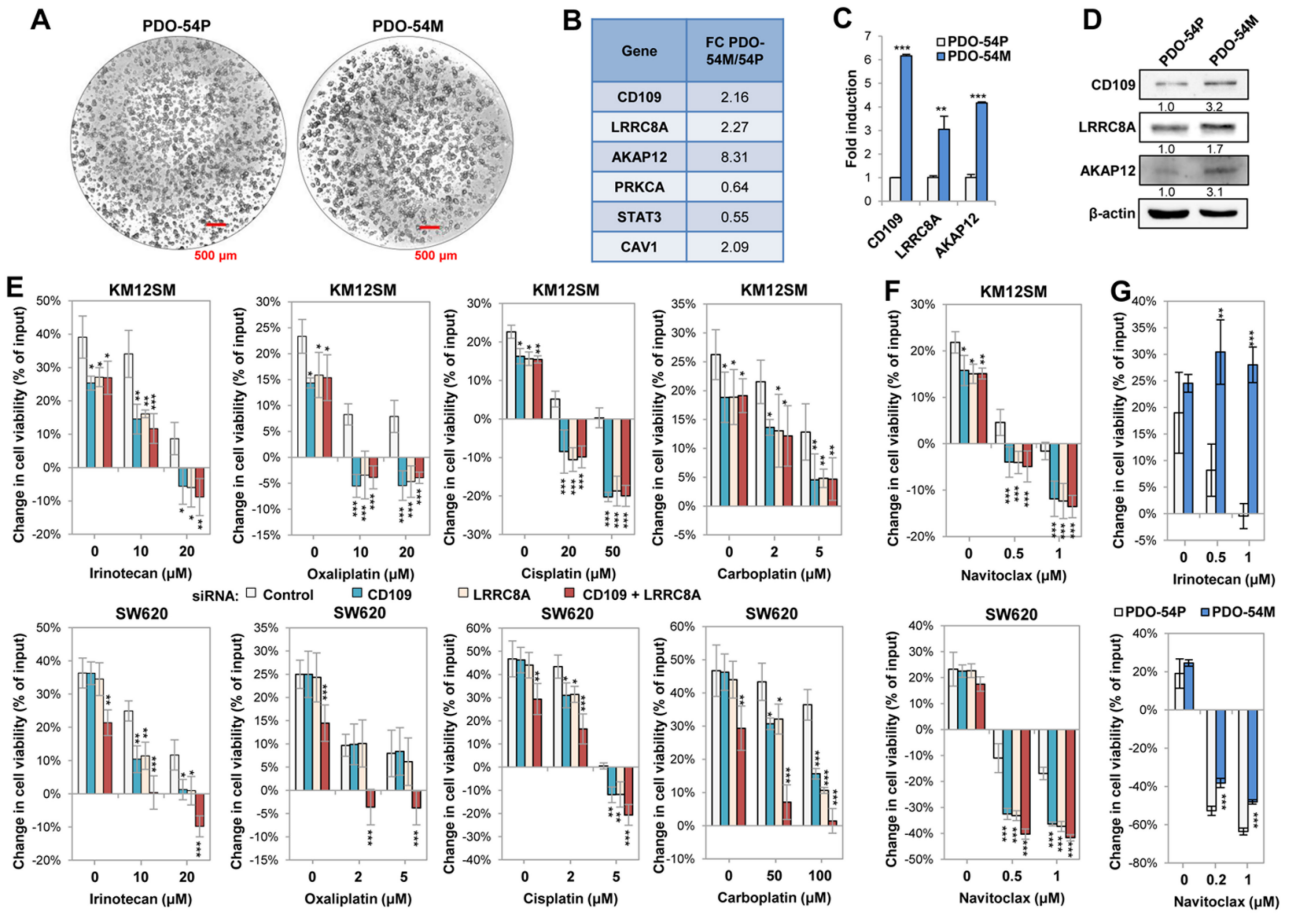
** The protein complex CD109/LRRC8A/AKAP12 is overexpressed in a metastatic PDO and promotes chemoresistance.** (A) Representative images of the indicated PDOs. The red bar represents 0.5 mm. (B) Fold-change of the indicated genes after *CD109* silencing, according to transcriptomic analyses of PDO-54M and PDO-54P, (C) qPCR and (D) Western blot analyses of the indicated proteins in PDO-54P and PDO-54M organoids. Gene expression was significantly enhanced in the metastatic PDO (*, p < 0.05; **, p < 0.01; ***, p < 0.001). Band quantification is shown below each band. (E, F) KM12SM and SW620 were transfected with control, *CD109*- and/or *LRRC8A*-targeting siRNAs and subjected to cell viability assays (48h) in the presence of the indicated concentrations of chemotherapy drugs (E) or the senolytic Navitoclax (F). Cell viability was significantly decreased following the silencing of *CD109* and/or *LRRC8A* (*, p < 0.05; **, p < 0.01; ***, p < 0.001). (G) The PDOs were subjected to cell viability assays (48h) in the presence of the indicated drugs. Cell viability was significantly increased in the metastatic counterpart (**, p < 0.01; ***, p < 0.001). Overall, data are representative of three independent experiments.

Colonic PDOs are used to mimic CRC *in vitro* and get further insights into molecular mechanisms underlying tumorigenesis. In order to investigate the association of CD109, LRRC8A, and AKAP12 expression with drug resistance in advanced CRC, we used PDO-54P and PDO-54M, which were established from a primary tumor and its liver metastasis, respectively (**Fig. 6A**). A transcriptomic analysis, validated by qPCR and Western blot, confirmed that CD109, LRRC8A and AKAP12, together with caveolin 1 (CAV1), were upregulated in the metastatic PDO-54M compared with the primary PDO-54P, (**Fig. 6B-D**), supporting the observed correlation between CD109/LRRC8A/AKAP12 expression, metastasis and drug resistance.

Next, we investigated the potential synergy of CD109 with LRRC8A in chemoresistance in cell lines and PDOs. The silencing of either CD109 or LRRC8A caused a reduction in cell viability after treatment with CPT-11 (irinotecan), cisplatin and carboplatin in both cell lines (**Fig. 6E**), but not 5-Fluorouracil (5-FU) (**Fig. S11**). However, SW620 cells required double silencing to show high sensitivity to oxaliplatin (**Fig. 6E**). Dose-response analysis showed that KM12SM cells were more resistant to oxaliplatin and cisplatin than SW620 cells (10-20 µM *vs* 2-5 µM), but were more susceptible to carboplatin (**Fig. 6E**). These differences in the sensitivity to platinum salts might be explained by the different composition of the VRAC heteromers (LRRC8 A/B *vs* A/D), which might affect the uptake capacity of the channel, as observed in the patch-clamp recordings (**Fig. S7, S8**).

Given the upregulation of senescence on CD109 KD cells (**Fig. 2C, Fig. S4**), we tested the sensitivity to Navitoclax, a senolytic agent. CD109 or LRRC8A silencing caused a drastic reduction in the cell lines viability following treatment with Navitoclax (**Fig. 6F**). Since the potential association of PDO-54M with irinotecan resistance, we assessed the role of CD109 and LRRC8A in mediating resistance to irinotecan and navitoclax in both PDOs. The results indicate a higher irinotecan resistance in the metastatic PDO, and high sensitivity to navitoclax in both PDOs, particularly in the primary PDO (**Fig. 6G**). Collectively, these results confirm the implication of CD109/LRRC8A/AKAP12 in drug resistance, and the potential benefits of treatment with senolytics.

### CD109 knockdown improves survival to liver metastasis

**Figure 7:**
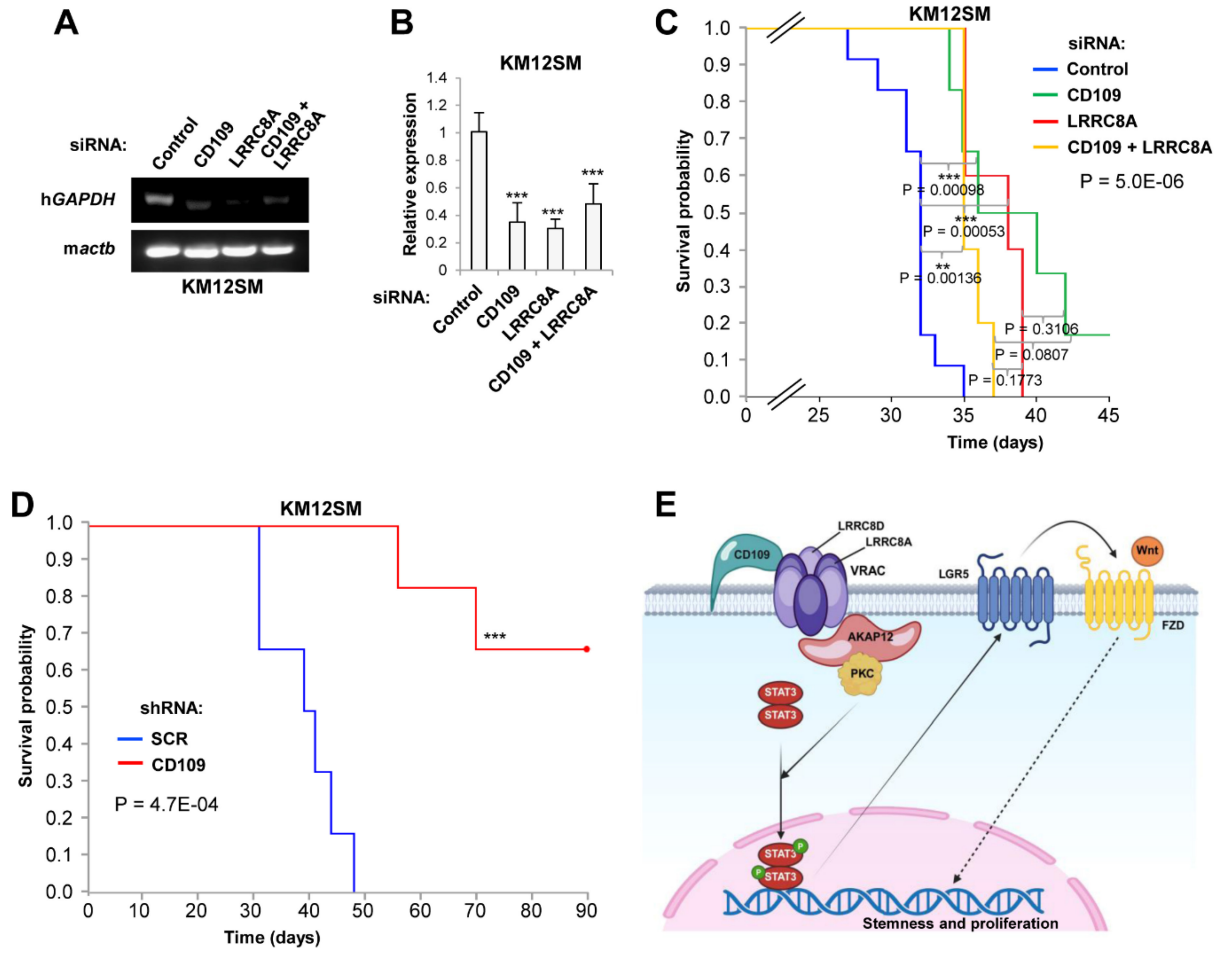
** CD109 promotes metastatic colonization of colorectal cancer cells.** (A, B) Metastatic KM12SM cells were transfected with control, *CD109*- and/or *LRRC8A*-targeting siRNAs and inoculated in the spleen of mice. After 72 h, mice were euthanized and the mRNA was isolated from the liver and subjected to RT-PCR (A) and qPCR (B) to amplify human *GAPDH*. Mouse *Actb* gene was amplified as control. Liver homing was significantly inhibited after *CD109* and/or *LRRC8A* gene silencing (***, p < 0.001). (C) Kaplan-Meier analysis of NSG mice inoculated in the spleen with the same KM12SM transfectants as in A. Log rank p-values for each pair of conditions, as well as the global one, are shown. (D) Kaplan-Meier analysis of Swiss nude inoculated in the spleen with SCR or shCD109 KM12SM stable transductants. The silencing of *CD109* and/or *LRRC8A* significantly enhanced mouse survival (**, p < 0.01; ***, p < 0.001). (E) Schematic representation of the proposed signaling model. CD109 interacts with LRRC8A-containing VRAC channels and recruits AKAP12/PKCα to activate STAT3. Activated STAT3 translocates to the nucleus and induces *LGR5* expression, enhancing Wnt/FZD signaling and stemness programs. Dashed arrows denote indirect interactions. This model was generated with BioRender.

Finally, we explored the impact of CD109 and LRRC8A silencing in liver metastasis and survival using KM12SM-derived mouse models, given the low metastatic potential of SW620 cells. To assess liver homing, CD109 and/or LRRC8A-silenced KM12SM cells were injected into the spleens of NSG mice. At 96h post-inoculation, human Glyceraldehyde-3-Phosphate Dehydrogenase (GAPDH) levels in liver tissue revealed that CD109, LRRC8A, and double knockdown cells exhibited reduced homing capacity (**Fig. 7A, B**). Kaplan-Meier survival analyses showed that silencing either CD109 or LRRC8A led to a modest but consistent extension in survival in NSG mice, while the double knockdown showed a similar effect (**Fig. 7C**). Stable silencing of CD109 did not increase further survival in NSG mice (data not shown). Strikingly, when CD109-silenced cells were injected in Swiss nude mice, which maintain the natural killer (NK) cytotoxic activity, survival was significantly longer (**Fig. 7D**). However, interferon γ levels in inoculated Swiss nude mice were below the limit of quantification, preventing further assessment of NK cell involvement. Overall, these findings are consistent with a role for CD109 in regulating STAT3 activation, inflammation, and metastatic progression.

## Discussion

Treatment of advanced CRC remains a significant clinical challenge, largely due to the persistence of therapy-resistant CSCs. Identifying molecules implicated in stemness maintenance is therefore critical for overcoming drug resistance. CD109, originally described as a negative regulator of TGFβ signaling 31, has emerged as a multifaceted player in cancer progression. Functionally, loss of CD109 reduced STAT3 activation, cell proliferation, and LGR5 expression, consistent with impaired Wnt signaling, while increasing sensitivity to JAK/STAT inhibitors. Notably, the inhibitory effects of CD109 knockdown on signaling pathways were more pronounced in LGR5^high^ metastatic KM12SM cells compared to the mesenchymal-like SW620 cells, consistent with previous reports indicating that LGR5^high^ populations display greater plasticity and play a critical role in metastatic progression in CRC 41. Mechanistically, we identified a physical and functional interaction between CD109 and LRRC8A. This complex regulates STAT3 activation via a downstream pathway involving the scaffolding protein AKAP12 and the PKCα kinase. Either AKAP12 silencing or pharmacological inhibition of PKCα decreased STAT3 phosphorylation and reduced LGR5 expression, supporting a CD109/LRRC8A/AKAP12/PKCα axis that regulates STAT3-driven stemness, Wnt signaling and, consequently, CSC maintenance (**Fig. 7E**). Importantly, knockdown of CD109 and LRRC8A significantly increased survival in mice with liver metastases, highlighting the therapeutic relevance of this pathway.

High CD109 expression was preferentially associated with the CRIS-B subtype, which is characterized by high TGFβ activity, EMT and poor prognosis 24. This subtype is also enriched in inflammatory phenotype hallmarks (IL6/JAK-STAT, TNFα/NF-κB, interferon-γ) associated with TGFβ activation 24. These features closely overlap with the gene expression signatures upregulated upon CD109 silencing, which indicate increased TGFβ activity. Together, the correlation between CD109 and TGFβ expression, along with our transcriptomic data, supports the capacity of CD109 to regulate TGFβ activity in CRC, consistent with previous observations in cancer cells and fibroblasts 8. Moreover, although the canonical TGFβ pathway is largely inactive in CRC metastatic cells, our data suggest that CD109 continues to modulate TGFβ signaling through non-canonical pathways, including MAPK, NF-κB, and Rho-GTPases. Consistently, the loss of CD109 promoted EMT through distinct mechanisms depending on the cellular context. In line with this, CD109 depletion has been shown to enhance TGFβ signaling, EMT, migration, and invasion in squamous cell carcinoma 42. Collectively, these findings support a model in which CD109 restrains TGFβ signaling to facilitate mesenchymal-epithelial transition during metastatic colonization, thereby balancing its pro-invasive and anti-proliferative effects.

The interaction of CD109 with LRRC8A but not with LTBP1 or GP130 as previously reported in lung cancer 10 and GBM 17, respectively, suggests a context-dependent CD109 interactome. The functional relevance of CD109 in VRAC activity was confirmed following its depletion, which resulted in a significant reduction in channel currents under both hypo- and hypertonic conditions. Our data support a key role for CD109 in the efficient activation and/or maintenance of LRRC8A-containing VRAC mediated currents. In addition to LRRC8A, CD109 associates with LRRC8B in KM12SM and with LRRC8D in SW620, suggesting that VRAC composition in CRC may adapt to the environmental pressure, such as drug exposure, to modulate channel selectivity 43. Structurally, LRRC8A and LRRC8B share the Arg^103^ residue, which together with LRRC8D Phe^143^, form the narrowest region of the VRAC pore 44, which determines inhibitor binding and substrate specificity 45. These structural features may underlie differences in chemotherapeutic responses across CRC cells. Consistent with this, LRRC8 subunits have been differentially implicated in platinum drug sensitivity. So, LRRC8A expression has been linked to oxaliplatin resistance in CRC cells 46 and doxorubicin resistance in ovarian cancer 47 while incorporation of LRRC8D into VRAC increases the uptake of cisplatin and carboplatin and promotes apoptosis in breast cancer cells 45, 48. In line with these observations, we found higher VRAC activity and increased resistance to carboplatin in SW620 compared to KM12SM cells. In our data, the loss of CD109 and LRRC8A increased sensitivity to platinum salts and irinotecan, being more relevant in KM12SM, suggesting that CD109 regulates VRAC-dependent drug responses in a cell context-dependent manner influenced by subunit composition. In contrast, there is currently no clear evidence indicating that VRAC directly mediates 5-FU uptake or resistance. Therefore, neither CD109 nor LRRC8A silencing affects 5-FU sensitivity in this context.

Although LRRC8A was previously implicated in hepatocellular carcinoma growth via PKCα signaling 38, a direct mechanistic link has not been established. Our results support a model in which the CD109/LRRC8A/AKAP12 complex scaffolds PKCα to regulate STAT3 activation, which in turn controls LGR5 expression. Consistently, pharmacological inhibition of STAT3 with Stattic reduced both LGR5 expression and Wnt signaling activity, whereas LGR5 silencing did not affect STAT3 activation, indicating that STAT3 acts upstream of LGR5. Our findings are consistent with a previous report that identified the binding of a complex formed by IKKα and STAT3 to the LGR5 promoter for regulating its expression 40. So, we propose a model in which CD109/LRRC8A-dependent STAT3 activation lies upstream of, and regulates, LGR5-driven Wnt signaling. Interestingly, AKAP12 overexpression has been reported to increase the expression of stem cell markers such as CD133 and SOX2 in metastatic CRC cells 36. In agreement with this, we observed downregulation of these markers following CD109 silencing.

Our proposed protein-protein interaction model is further sustained by clinical and PDOs gene expression data. We observed concurrent overexpression of CD109, LRRC8A, and AKAP12, which correlates with poor prognosis across multiple CRC transcriptomic datasets and with elevated expression in metastatic PDO-54M compared to primary PDO-54P. These results are consistent with publicly available data from the Human Protein Atlas, KM-Plotter, and GEPIA webtools (data not shown), as well as with a previous IHC-based study reporting LRRC8A overexpression in CRC 49. Unfortunately, no suitable antibodies were found for IHC validation of AKAP12. Notably, CAV1 overexpression was also observed in PDO-54M (**Fig. 6A**). CAV1 has been reported to interact with LRRC8A to regulate VRAC activity 50, and with PKCα to promote STAT3 activation either by retaining JAK/STAT components 51 or by facilitating receptor complex formation at the plasma membrane 52. CAV1 may help to spatially organize the CD109/LRRC8A/AKAP12 complex, thereby enhancing PKCα/STAT3 activity. A limitation of this study is that the coordinated upregulation of CD109/LRRC8A/AKAP12 with CAV1 expression was observed in a single paired PDO model (PDO-54). Further data on additional PDOs will be required to confirm the generality and clinical relevance of this finding.

STAT3 is a central hub for tumor-promoting inflammation and has been shown to drive the expression of IL-6, IL-10, and VEGF, which contribute to an immunosuppressive microenvironment 53, 54. In addition, Wnt/STAT3-active cells promote immunosuppression by recruiting Foxp3^+^ Treg cells to the metastatic niches 55. Based on these observations, it is plausible that CD109 may influence the tumor immune microenvironment. In line with this possibility, we observed modest but significant differences in survival in highly immunocompromised NSG mice upon CD109 depletion. However, apparent differences between double and single knockdowns were not statistically significant and were considered not relevant. In contrast, more pronounced effects were detected in the more immunocompetent Swiss mouse model. Furthermore, CD109 silencing was associated with increased expression of TNFα and interferon-related signaling pathways, which have been linked to enhanced cytotoxic immune responses, including NK cell activity. However, as interferon-γ could not be reliably detected in the nude mouse models, these observations should be interpreted with caution. Additional studies are required to directly assess the contribution of immune cell populations to the observed results.

Altogether, our results integrate previously fragmented observations into a coherent model, where the CD109/LRRC8A/AKAP12/PKCα complex regulates a STAT3-Wnt signaling axis that drives CSC maintenance, EMT plasticity, and drug resistance in CRC. High co-expression of these components correlates with poor prognosis across datasets and human PDOs. Targeting STAT3 activation in CRC has shown promise in clinical trials, given its association with EMT, stemness and drug resistance 56, 57. Here, we propose that the alternative therapeutic targeting of CD109 or its downstream effectors could not only suppress STAT3-driven metastasis but also enhance sensitivity to JAK/STAT inhibitors and standard chemotherapy. Given its membrane localization and secretion, CD109 is an attractive candidate for antibody-based interventions. In summary, the CD109 pathway supports the survival of a therapy-resistant, stem-like cell population in advanced cancer stages and represents a promising therapeutic strategy in advanced CRC.

## Supplementary Material

Supplementary figures.

Supplementary table 1.

Supplementary table 2.

## Data Availability

The datasets generated during the current study are available from the corresponding author on reasonable request.
