# Supplementary material for: CD109 Drives Stemness and Chemoresistance in Colorectal Cancer via LRRC8A/AKAP12/PKCα-Mediated STAT3 Activation and WNT Signaling
Source: Int J Biol Sci. 2026 Jun 4;22(11):6064–83. doi: 10.7150/ijbs.127856 (PMC13282776; doi:10.7150/ijbs.127856)
Supplement: Supplementary file 1 — Supplementary figures. [file ijbsv22p6064s1.pdf]

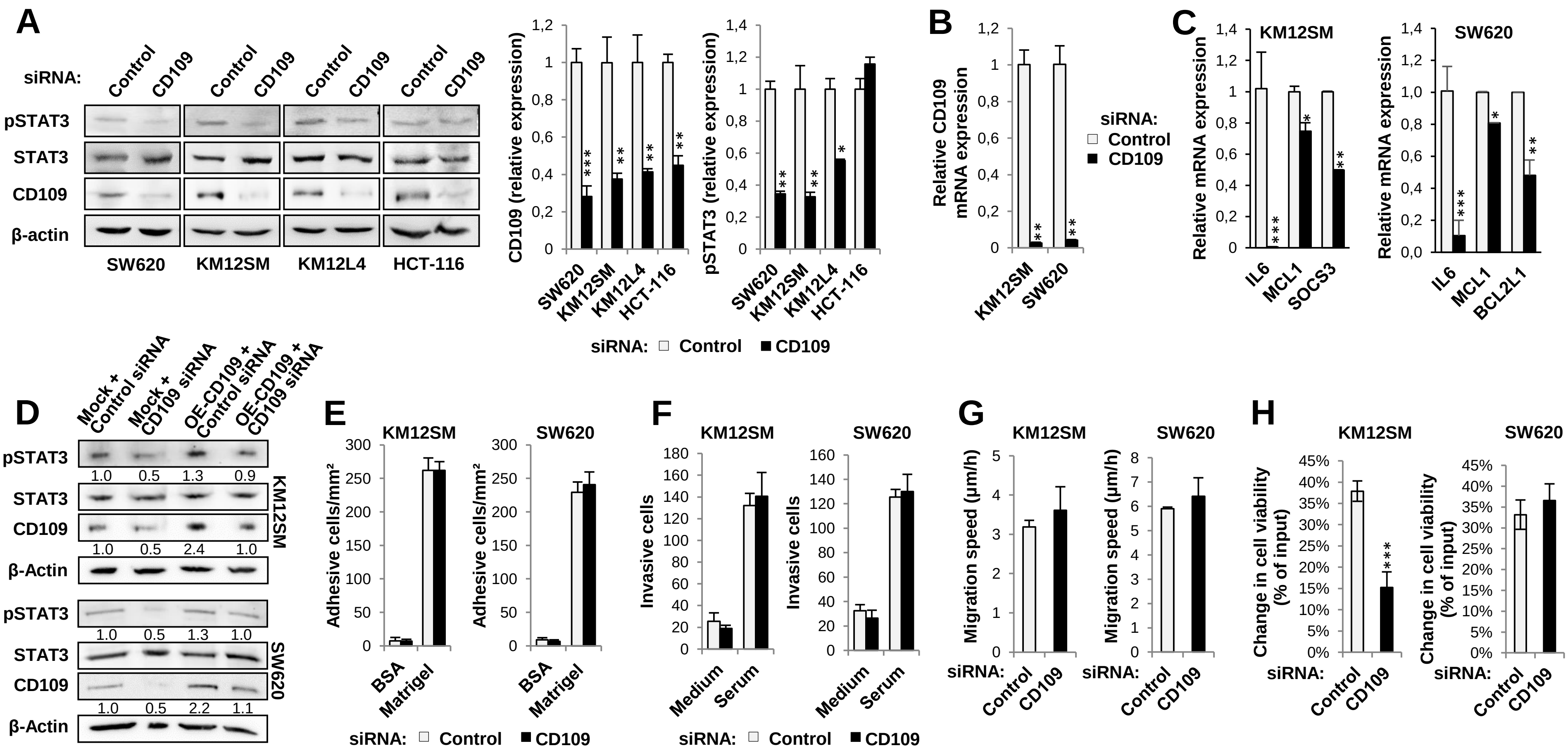

Figure S1. CD109 promotes the activation of STAT3 in colon cancer cells. (A) Western blot analyses of the indicated proteins were performed with protein extracts from the indicated cell lines transfected with control or CD109-targeting siRNAs. The transfection of CD109 siRNAs caused a significant decrement in CD109 and p-STAT3 expression (*, $p < 0.05$; **, $p < 0.01$; ***, $p < 0.001$). The same KM12SM and SW620 transfectants were analyzed by qPCR to confirm the downregulation in CD109 expression (B), as well as the downregulation in STAT3 targets (C). The transfection of CD109 siRNAs caused a significant decrement in CD109 and STAT3 target mRNA expressions (*, $p < 0.05$; **, $p < 0.01$; ***, $p < 0.001$). (D) KM12SM and SW620 cells were transfected with empty vectors (Mock) or vectors coding to CD109 (OE-CD109) and with Control or CD109-targeting siRNAs. After 48 h cell lysates were analyzed by Western blot to detect the indicated proteins. Band quantification is shown below each lane. The same transfectants as in A were subjected to cell adhesion (E), cell invasion (F), cell migration (G) and cell viability (H) assays. The transfection of CD109 siRNAs provoked a significant inhibition in cell proliferation (***, $p < 0.001$).

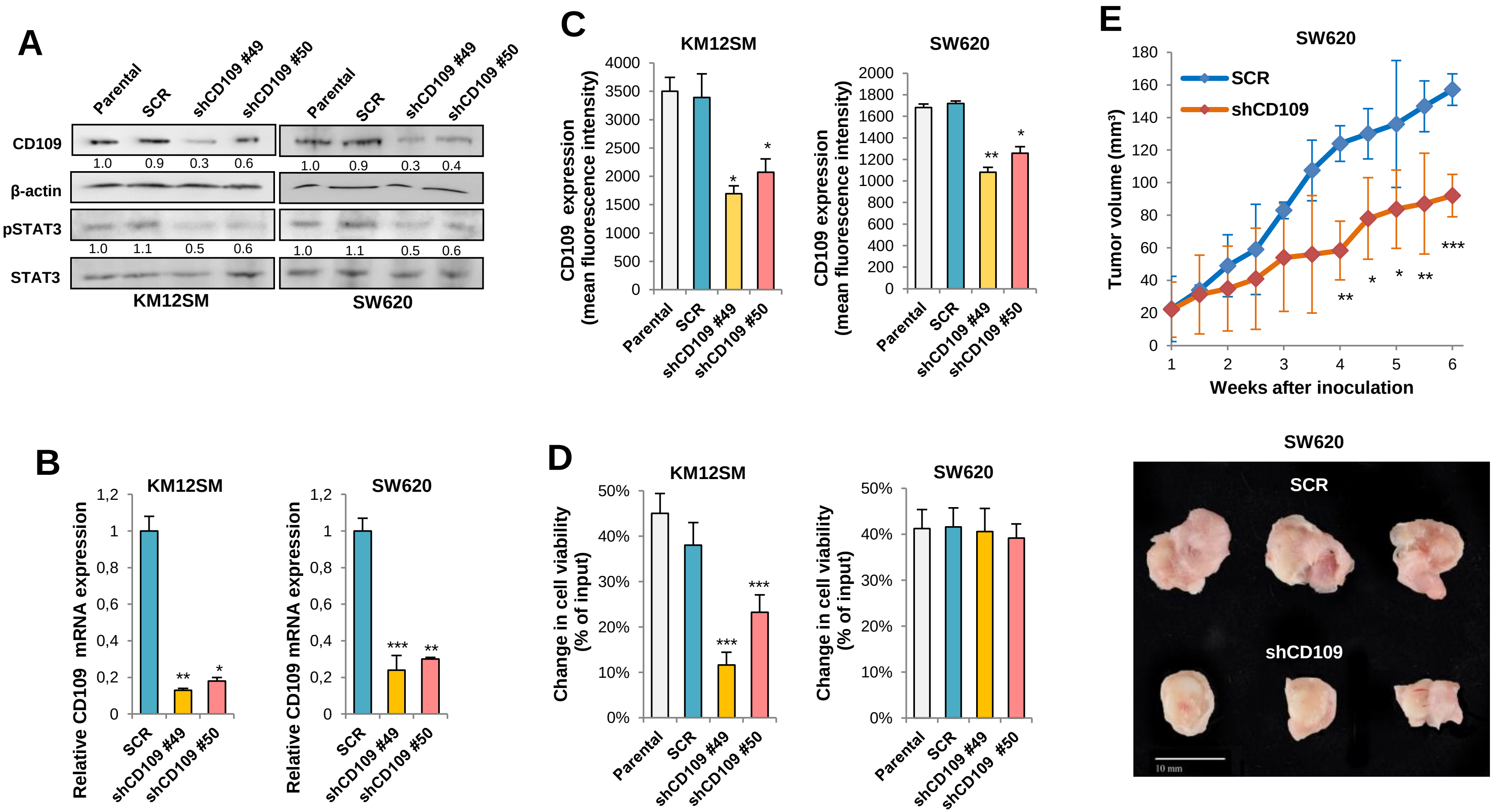

Figure S2. Generation of CD109 silenced stable transfectants. KM12SM and SW620 cells were infected with lentiviral particles containing two different shRNAs against CD109 (“shCD109 #49” and “shCD109 #50”) or a scramble (“SCR”) shRNA. Infected cells (GFP+) were selected in a sorter. After 2 rounds of selection, cells were lysed and the extracts subjected to western blot analysis to detect the knocking down expression of CD109 and phospho-STAT3. Band quantification is shown below each lane. Quantitative PCR (B) and flow cytometry (C) analysis of the same stable transfectants and the parental cell lines. The stable shCD109 transfectants showed a significant reduced expression of CD109 mRNA and of CD109 protein in the cell surface (*, $p < 0.05$; **, $p < 0.01$; ***, $p < 0.001$). (D) Cell viability assays of the same transfectants and parental cell lines. Cell viability was significantly reduced in the shCD109 transfectants (***, $p < 0.001$). (E) The indicated stable transfectants were inoculated subcutaneously in NSG mice. Tumor volume was measured twice per week. Representative images of xenografts at the end of the experiment are shown. The stable shCD109 transfectants showed a significant reduction in tumor growth compared to SCR transfectants (*, $p < 0.05$; **, $p < 0.01$; ***, $p < 0.001$).

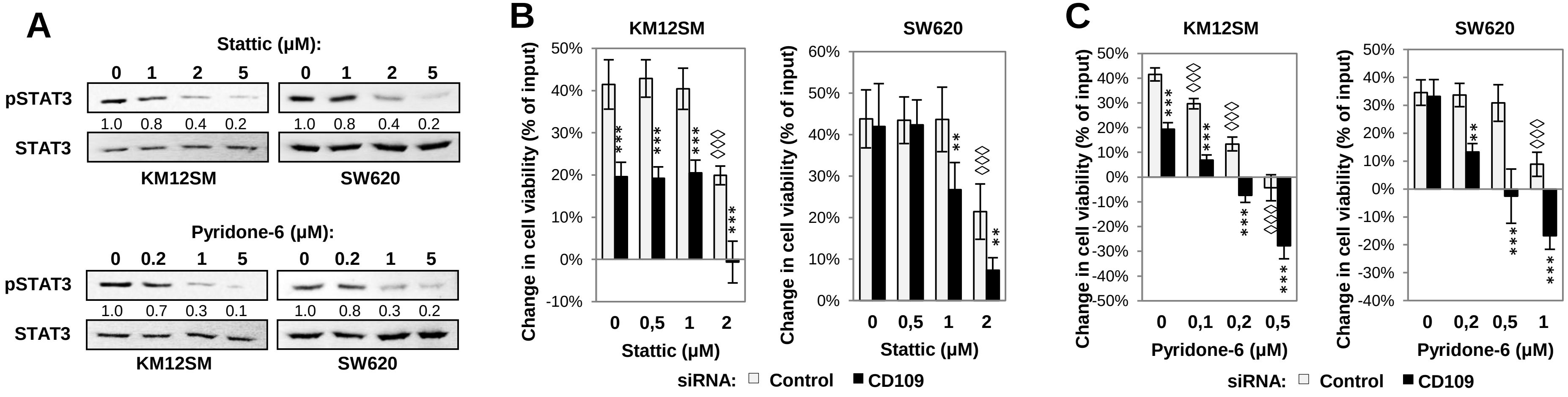

Figure S3. CD109 promotes colon cancer cell survival by enhancing the activation of STAT3. (A) KM12SM and SW620 cells were exposed to the indicated concentrations of the STAT3 inhibitors, Stattic and Pyridone-6 for 20 h, and lysed, and the protein extracts analyzed by Western blot to detect the indicated proteins. P-STAT3/total STAT3 ratios are shown. (B, C) The indicated cell lines were transfected with control or CD109-targeting siRNAs and subjected to cell viability assays in the presence of the indicated concentrations of Stattic (B) or Pyridone-6 (C). Cell viability was significantly reduced by CD109 silencing (**, $p < 0.01$; ***, $p < 0.001$) or the presence of the indicated inhibitors (◊◊◊, $p < 0.001$).

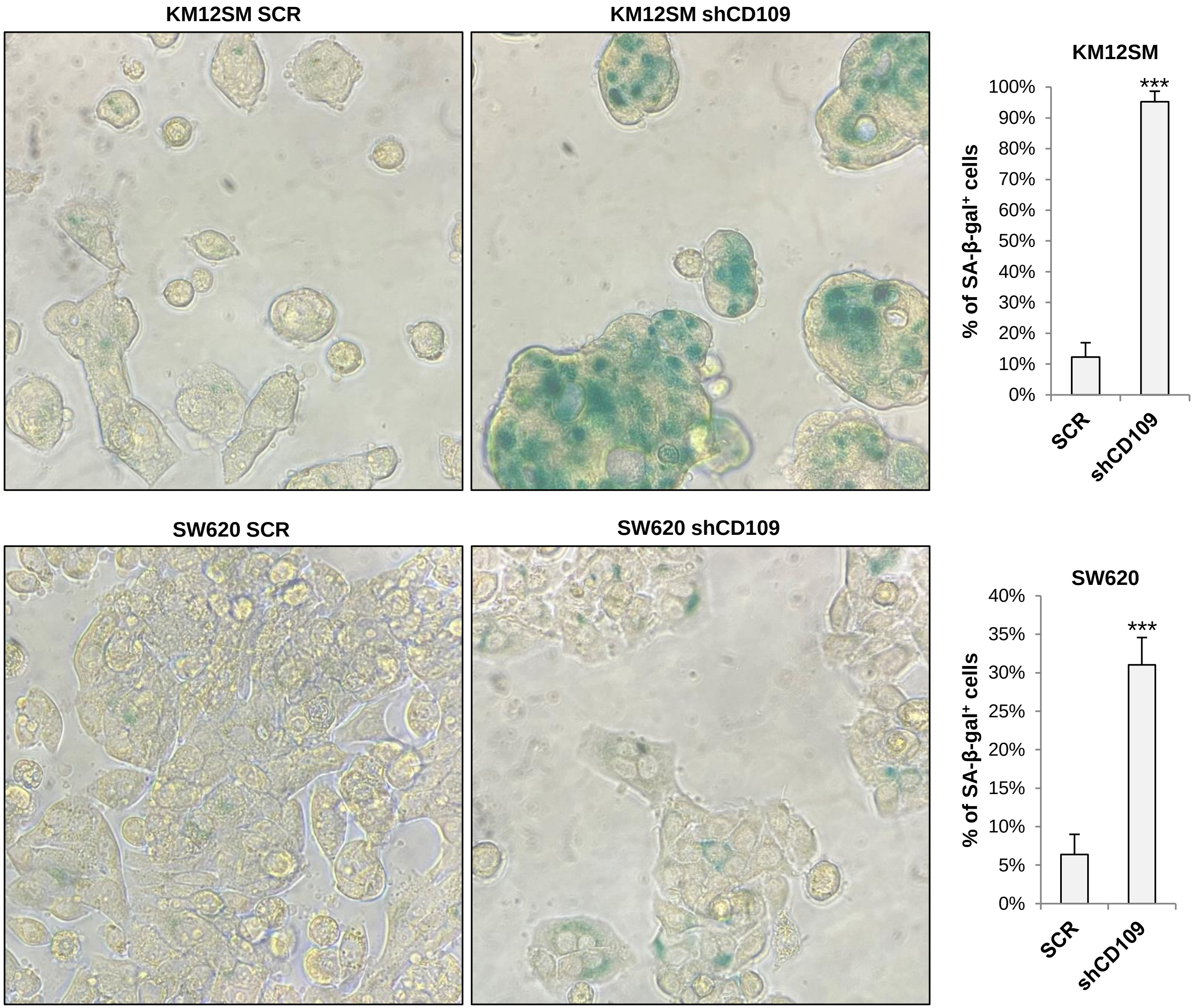

Figure S4. CD109 silencing makes colon cancer cells susceptible to senescence-inducing compounds. SCR and shCD109 KM12SM and SW620 cells were exposed to the senescence-inducing compound Palbociclib (5 μM) for 7 days. Then cells were fixed and incubated with an X-Gal staining solution. Representative images of the activity of senescence-associated-β-galactosidase (which appears with green color) are shown. Quantification of the images was performed using ImageJ, showing a significant increment in the percentage of CD109 silenced cells staining positive (***, $p < 0.001$).

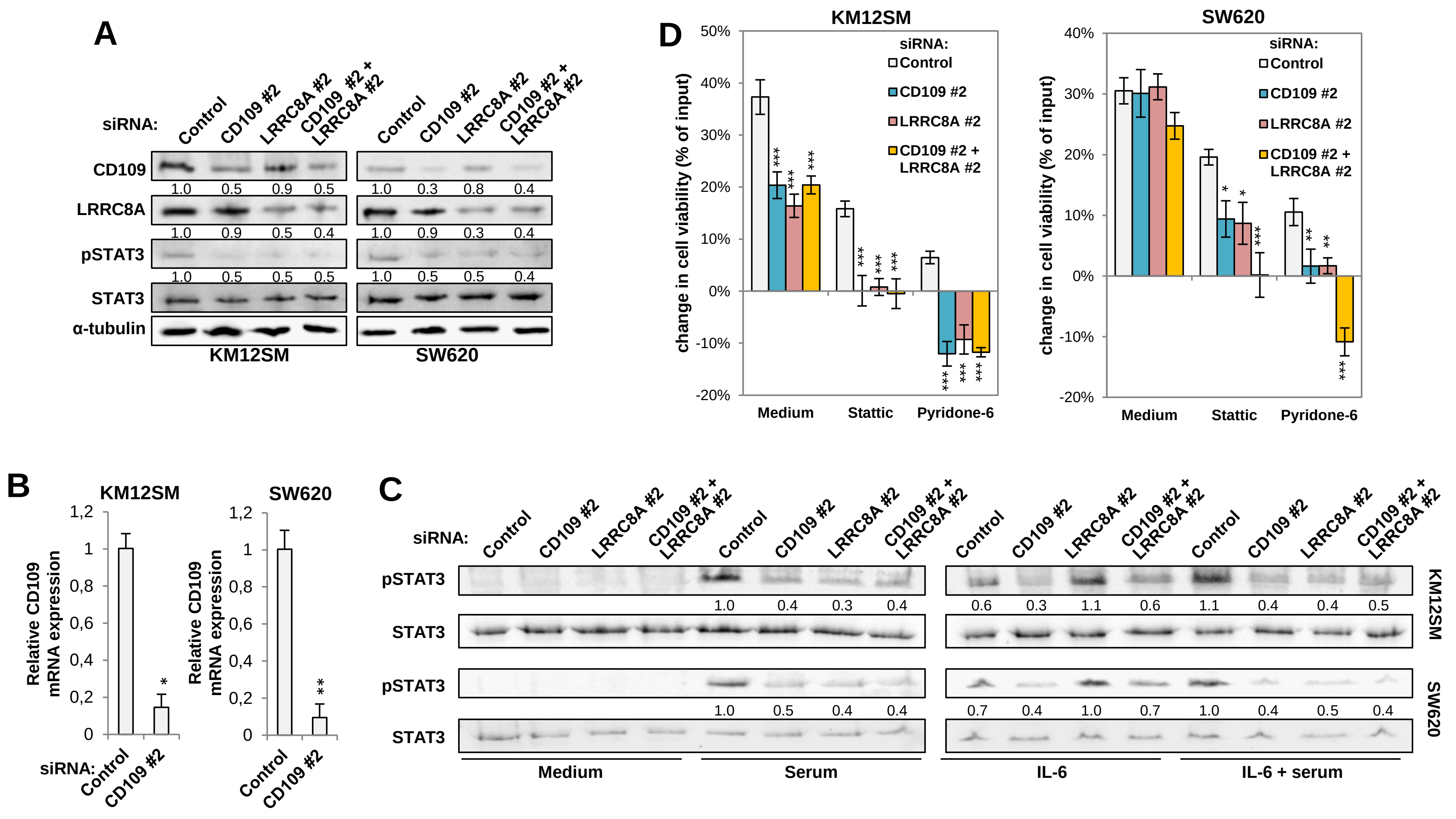

Figure S5. The protein complex CD109/LRRC8A promotes colon cancer cell survival by enhancing the activation of STAT3. (A) KM12SM and SW620 cells were transfected with the indicated siRNAs and lysed. The extracts were analyzed by Western blot to detect the indicated proteins. Band quantification is shown below each band. (B) Quantitative PCR analyses of CD109 expression levels in cells transfected with Control or CD109 #2 siRNAs. (C) The same transfectants as in A were incubated in serum-free medium for 3 h and exposed to IL-6 (10 ng/mL) and/or serum (10%) for 15 min. The extracts were analyzed by Western blot to detect the indicated proteins. Band quantification is shown below each band. D) The same transfectants as in A were subjected to cell viability assays in the presence of Stattic or Pyridone-6. Cell viability was significantly reduced by CD109 and/or LRRC8A silencing (*, $p < 0.05$; **, $p < 0.01$; ***, $p < 0.001$).

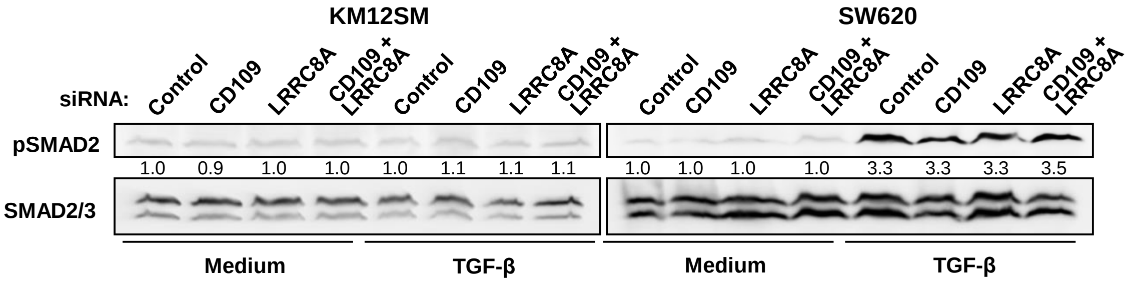

Figure S6. CD109 does not affect TGF-β signaling in metastatic colon cancer cells. The indicated transfectants were kept in starving, exposed to TGF-β1 (5 ng/mL, 1 h) and lysed. The protein extracts were analyzed by Western blot to detect the indicated proteins. The ratios between phosphorylated and total proteins are shown between the lanes.

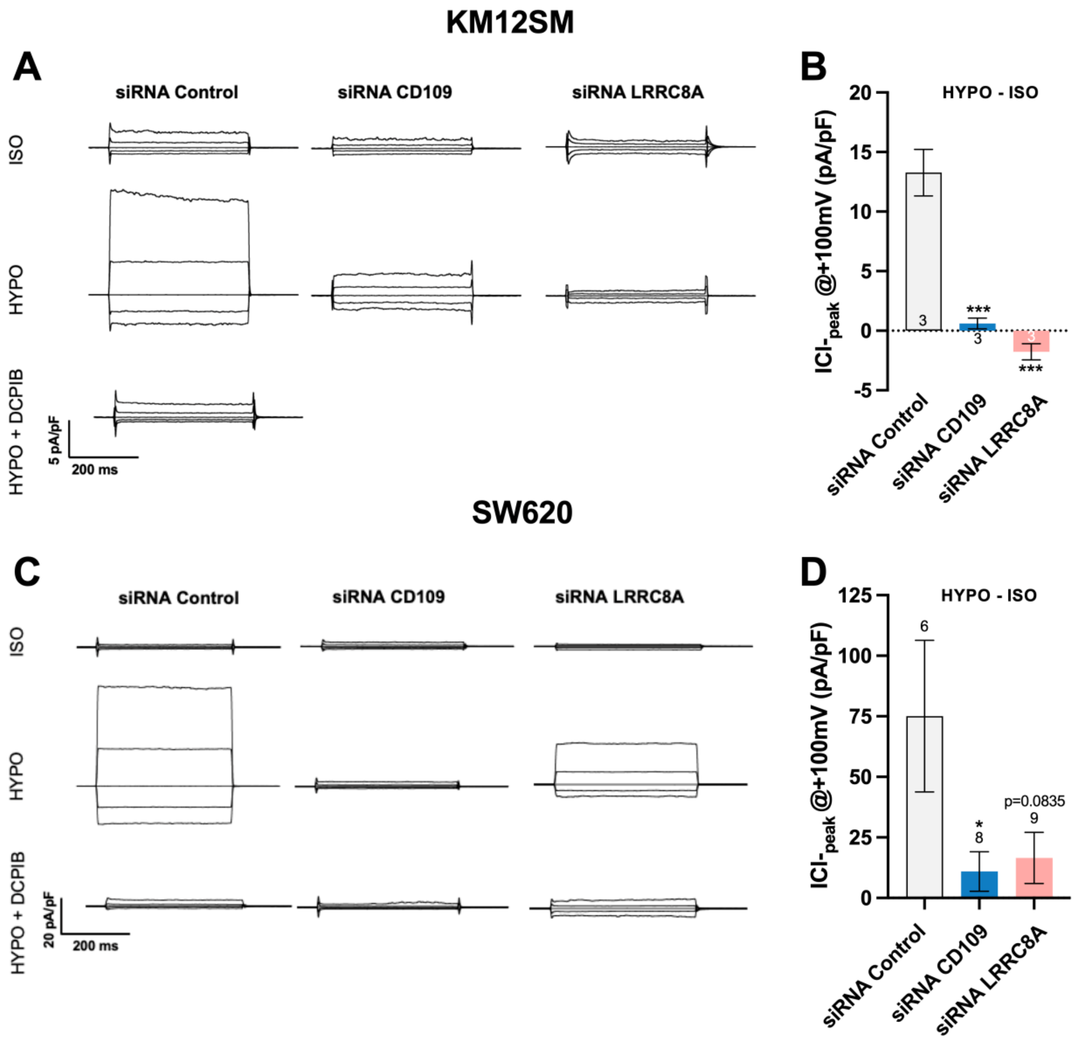

Figure S7. Hypotonicity-induced VRAC activity is reduced by CD109 or LRRC8A knockdown in the CRC metastatic cell lines. (A, C) Representative chloride current traces recorded in KM12SM (A) and SW620 (C) cells transfected with control, CD109-targeting, or LRRC8A-targeting siRNAs, as indicated. Shown currents were recorded under isotonic conditions immediately before hypotonic perfusion (ISO), after 10 min of hypotonic perfusion (HYPO), and following addition of the VRAC inhibitor DCPIB (40 µM) under hypotonic conditions (HYPO + DCPIB). Cells were held at 0 mV and pulsed from −100 mV to +100 mV in 50-mV increments. (B, D) Mean hypotonicity-induced VRAC activity in KM12SM (B) and SW620 (D) cells, calculated as the difference between the peak current density measured at +100 mV after 10 min of hypotonic perfusion and that recorded immediately before the onset of hypotonic perfusion (HYPO − ISO). The knockdown of CD109 or LRRC8A significantly (*, $p < 0.05$; ***, $p < 0.001$) reduced hypotonicity-induced VRAC currents.

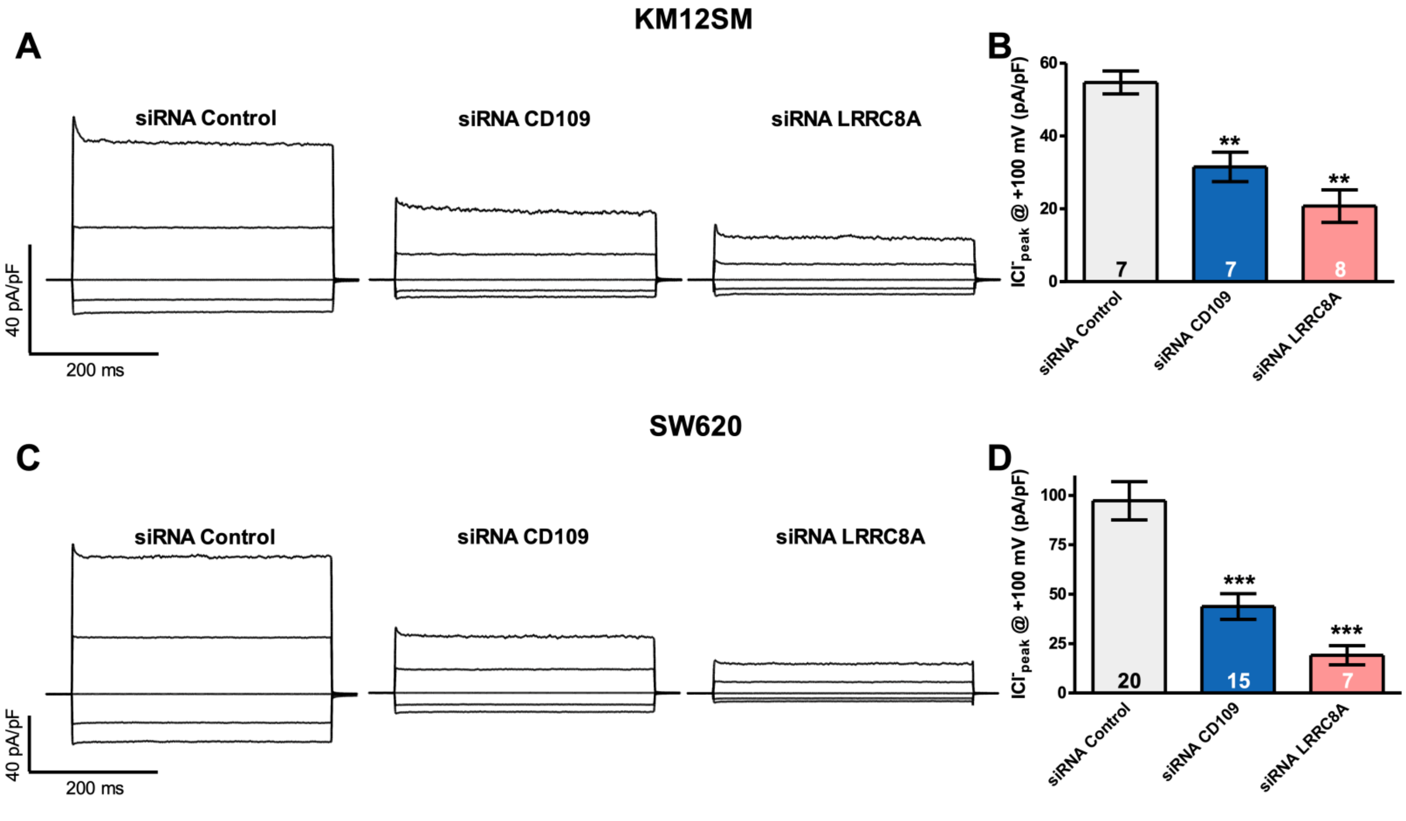

Figure S8. VRAC activity induced by intracellular dialysis with a hypertonic, low-ionic-strength solution is significantly reduced by CD109 or LRRC8A knockdown in the CRC metastatic cell lines KM12SM and SW620. (A, C) Representative families of chloride currents recorded 2 min after establishment of the whole-cell configuration in KM12SM (A) and SW620 (C) cells transfected with control, CD109-targeting, or LRRC8A-targeting siRNAs, as indicated. Cells were bathed in isotonic external solution and dialyzed with a hyperosmotic internal solution of low ionic strength (IS ~ 0.06 M). Cells were held at 0 mV and pulsed from −100 mV to +100 mV in 50-mV increments. (B, D) Maximal mean current densities measured in KM12SM (B) and SW620 (D) cells under the experimental conditions shown in A and C, respectively. Number of tested cells are shown inside each bar. Maximal mean current densities were significantly reduced after CD109 or LRRC8A silencing (**, $p < 0.01$; ***, $p < 0.001$).

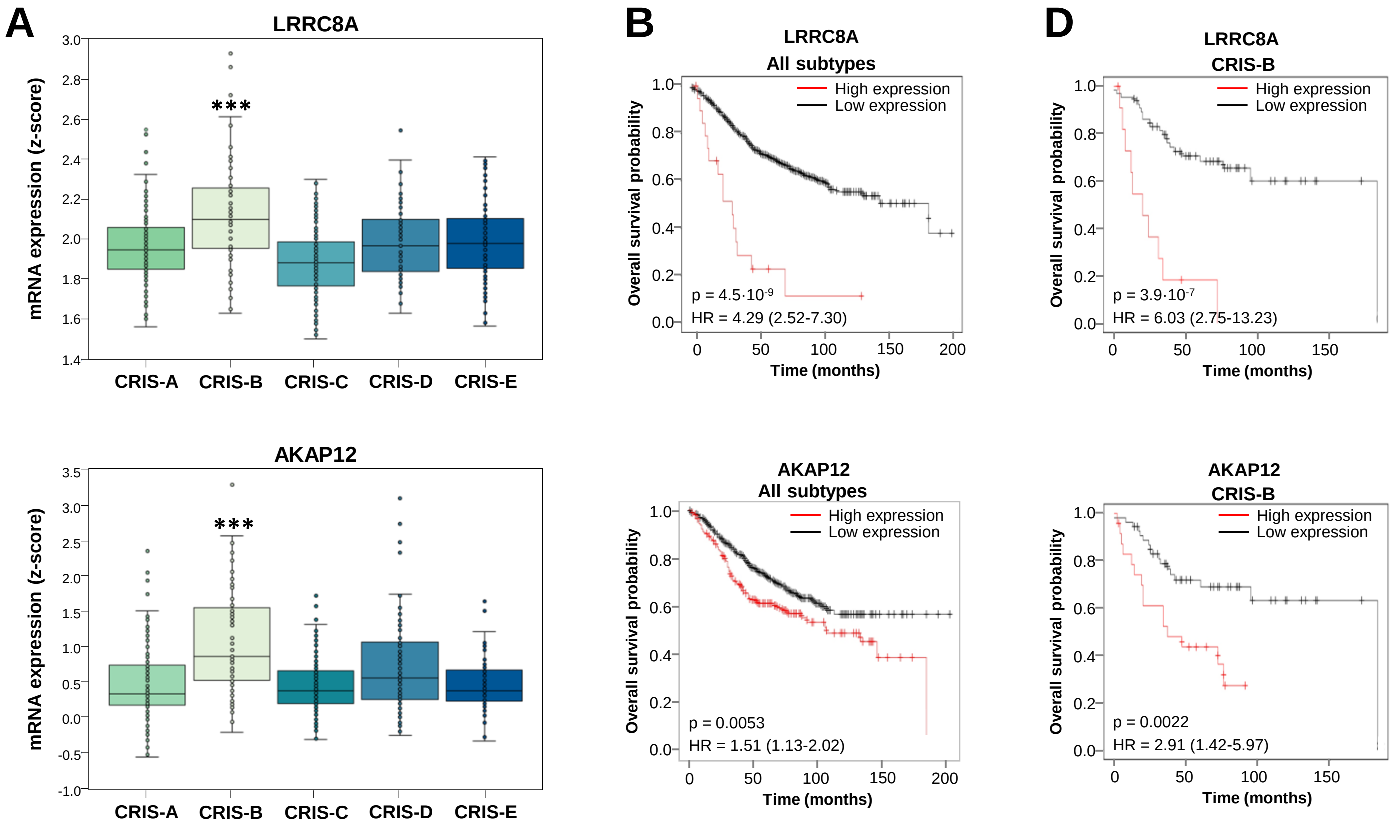

C

Overall survival p value	CRIS-A	CRIS-B	CRIS-C	CRIS-D	CRIS-E
**LRRC8A**	0.017	3.9E-07	0.13	0.013	0.026
**AKAP12**	0.016	2.2E-03	0.051	0.15	0.061

Figure S9. LRRC8A and AKAP12 are overexpressed in CRIS-B colon cancer subtype. (A) Expression z-score values of the indicated genes by CRIS subtype in GSE39582 colon cancer cohort. The expression of the indicated genes was significantly enhanced in CRIS-B subtype (***, $p < 0.001$). (B) Kaplan-Meier overall survival analysis of colon cancer patients in the same database according to the expression of the indicated genes. Log-rank test p values and Cox regression model HR are shown inside each graph. (C) Log-rank test p values of overall survival of colon cancer patients according to the expression of the indicated genes in the different Colorectal Cancer Intrinsic Subtypes (CRIS). (D) Kaplan-Meier overall survival analysis of CRIS-B colon cancer patients.

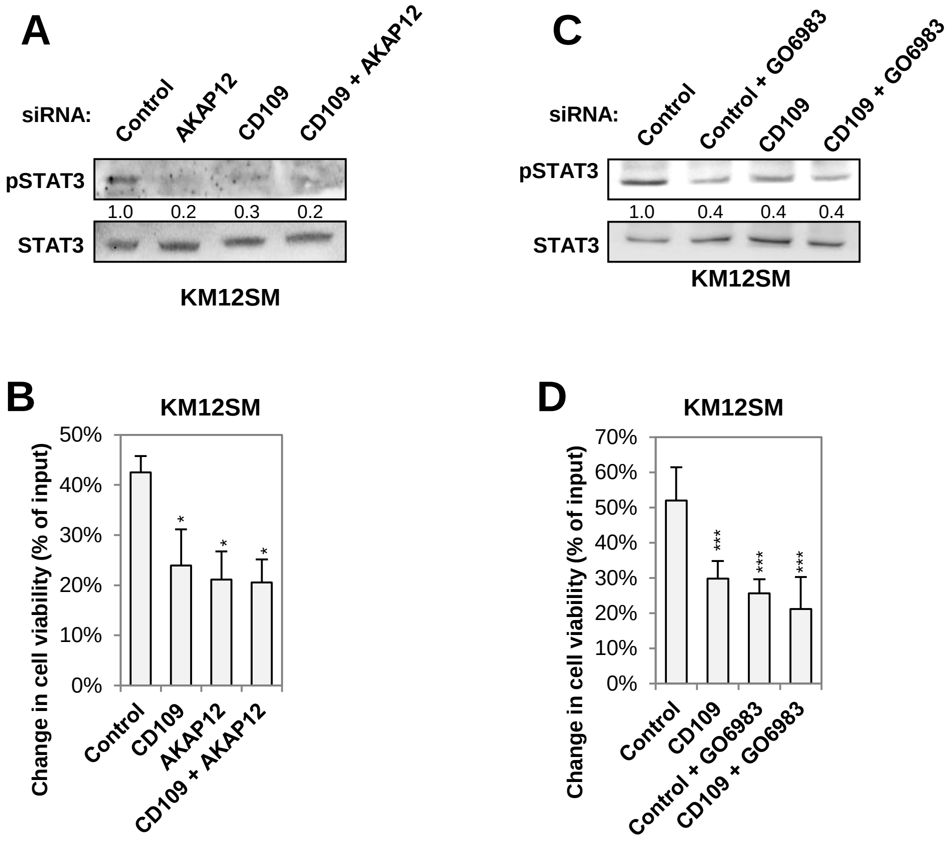

Figure S10. STAT3 activation and cell viability induced by CD109 are dependent of AKAP12 expression and PKC activity. (A) Western blot analysis of KM12SM cells previously transfected with control, AKAP12 and/or CD109-targeting siRNAs Band quantification is shown below the lane. (D) Cell viability assay of the same treated transfectants. (C) Western blot analysis of KM12SM cells previously transfected with control or CD109-targeting siRNAs and treated with or without GO6983 (0.3 μM). (D) Cell viability assay of the same treated transfectants. Cell viability was significantly reduced in GO6983 treated cells, in AKAP12-, or in CD109-silenced cells (*, $p < 0.05$; ***, $p < 0.001$).

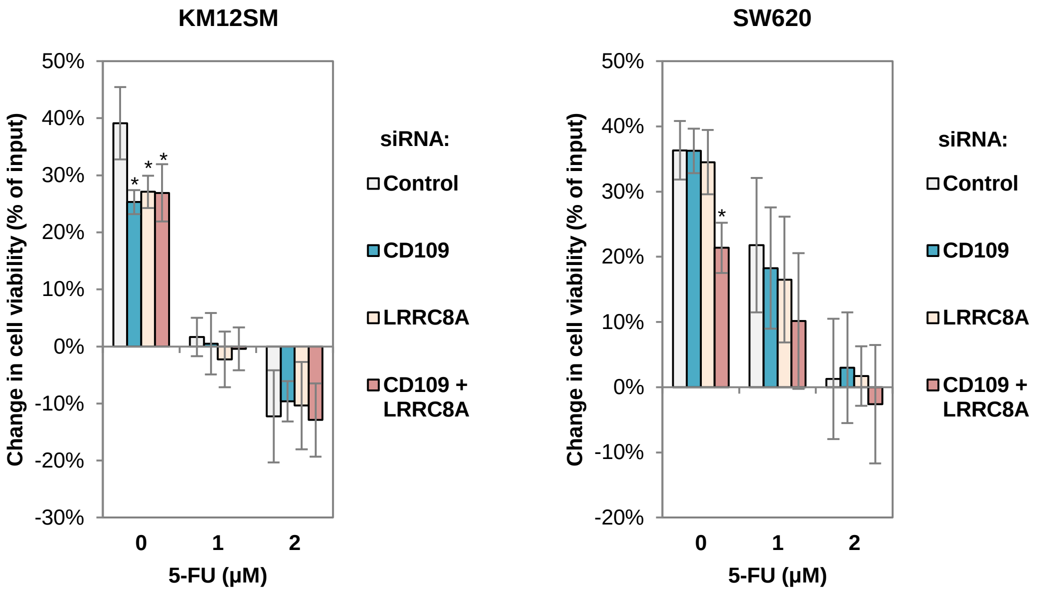

Figure S11. The protein complex CD109/LRRC8A does not affect chemoresistance to 5-Fluorouracil. KM12SM and SW620 were transfected with control, CD109- and/or LRRC8A-targeting siRNAs and subjected to cell viability assays in the presence of the indicated concentrations of 5-FU. Cell viability was significantly decreased by the silencing of CD109 and/or LRRC8A (*, $p < 0.05$).
